# Synthesis and Anti-Inflammatory Activity of Ferulic Acid-Sesquiterpene Lactone Hybrids

**DOI:** 10.3390/molecules29050936

**Published:** 2024-02-21

**Authors:** Xiyan Duan, Ning Liu, Ke Lv, Junqi Wang, Mingyue Li, Yanwei Zhang, Xiaoguang Huo, Shiqi Bao, Zhuo Shen, Xuemei Zhang

**Affiliations:** 1 School of Chemistry & Chemical Engineering, Henan University of Science and Technology, Luoyang 471003, China; 17513624203@163.com; 2 School of Nursing, Henan University of Science and Technology, Luoyang 471003, China; 9943548@haust.edu.cn; 3 The State Key Laboratory of Medicinal Chemical Biology & College of Chemistry, Nankai University, Tianjin 300071, China; 4 College of Pharmacy, Nankai University, Tianjin 300071, China; lmy1137534123@163.com; 5 Accendatech Company, Ltd., Tianjin 300384, China; zyw12130123@163.com (Y.Z.); huoxg0323@163.com (X.H.); shiqi.bao@accendatech.com (S.B.); shenzhuo@accendatech.com (Z.S.); xuemei.zhang@accendatech.com (X.Z.)

**Keywords:** parthenolide, micheliolide, ferulic acid, anti-inflammatory activity, acute lung injury

## Abstract

Acute lung injury (ALI) is a respiratory failure disease associated with high mortality rates in patients. The primary pathological damage is attributed to the excessive release of pro-inflammatory mediators in pulmonary tissue. However, specific therapy for ALI has not been developed. In this study, a series of novel ferulic acid-parthenolide (FA-PTL) and ferulic acid-micheliolide (FA-MCL) hybrid derivatives were designed, synthesized, and evaluated for their anti-inflammatory activities in vitro. Compounds **2**, **4**, and **6** showed pronounced anti-inflammatory activity against LPS-induced expression of pro-inflammatory cytokines in vitro. Importantly, compound **6** displayed good water solubility, and treatment of mice with compound **6** (10 mg/kg) significantly prevented weight loss and ameliorated inflammatory cell infiltration and edema in lung tissue, as well as improving the alveolar structure. These results suggest that compound **6** (((1a*R*,7a*S*,8*R*,10a*S*,10b*S*,*E*)-8-((dimethylamino)methyl)-1a-methyl-9-oxo-1a,2,3,6,7,7a,8,9,10a,10b-decahydrooxireno[2′,3′:9,10]cyclodeca[1,2-b]furan-5-yl)methyl (*E*)-3-(4-hydroxy-3-methoxyphenyl)acrylate 2-hydroxypropane-1,2,3-tricarboxylate) might be considered as a lead compound for further evaluation as a potential anti-ALI agent.

## 1. Introduction

Inflammation is a basic innate immune response to the disordered tissue homeostasis, such as allergens, pathogens, damaged cells, infection or tissue damage [1]. Many diseases arise from sites of infection and inflammation, including sepsis [2], atherosclerosis [3], diabetes [4], obesity and cancers [5]. Among the many diseases associated with inflammation, acute lung injury (ALI) is respiratory diseases with high mortality rates which is caused by pneumonia, pulmonary contusion, severe sepsis, gastroesophageal reflux, shock, transfusion, drug toxicity and acute pancreatitis [6]. In recent years, a series of studies have unveiled that blocking inflammatory response was considered as an effective strategy for treatment of ALI [7,8]. Although substantial progress has been achieved in the clinical therapeutics of ALI, the mortality of ALI still remains at a high level due to the lack of effective drug [9]. Thus, the discovery of novel and effective anti-inflammation agents for the treatment of ALI is highly desirable.

Parthenolide (PTL), a natural germacrane sesquiterpene lactone derived from Feverfew (*Tanacetum parthenium* L.), has demonstrated remarkable biological activities in cancer cells [10,11,12]. It has also attracted considerable interest for its potential therapeutic effects in inflammatory diseases [13]. PTL has been shown to ameliorate colon inflammation in a gut microbiota-dependent manner by improving Treg/Th17 balance, mediated through increased production of microbiota-derived SCFAs in intestinal mucosa [14]. In addition, PTL exhibits the ability to inhibit neuroinflammation, displaying potent anti-neuroinflammatory activity and neuroprotective effects [15,16]. Interestingly, in vivo studies have demonstrated that PTL attenuates bleomysion-induced pulmonary fibrosis by inhibiting the NF-κB/Snail signaling pathway [17].

**Figure 1:**
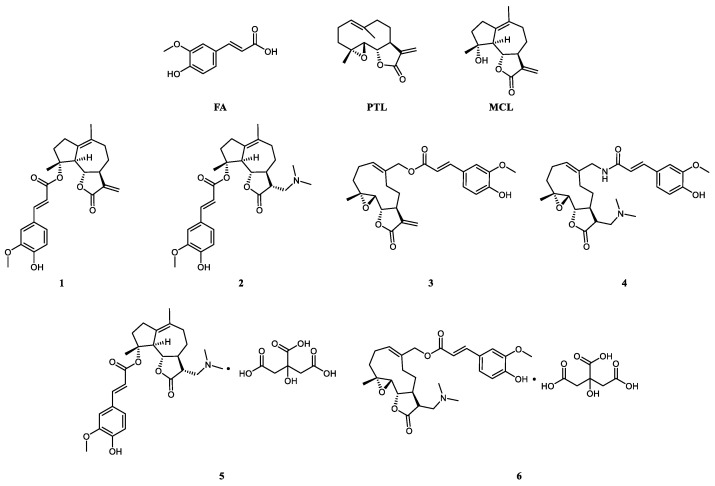
FA, PTL, MCL and their derivatives.

It is noted that PTL can be easily converted into micheliolide (MCL), a natural guaianolide sesquiterpene lactone which was isolated from Michelia compressa and Michelia champaca (Figure 1), in the presence of p-toluenesulfonic acid, with an excellent yield [18]. Compared with PTL, MCL has a more stable structure, improved pharmacokinetic profile in vivo and similar promising anti-inflammatory and anti-tumor properties [19,20,21]. Moreover, primary lung fibroblasts derived from eight patients with IPF and eight age-matched non-diseased controls (NDC) were treated with 0–10 µM ACT001, and results showed that ACT001 inhibited IL-6 but not IL-8 production in unstimulated fibroblasts [22]. Although tremendous advancements have been made in the field of sesquiterpenes lactones, poor water solubility limited its clinical application.

Ferulic acid (FA) is a naturally occurring phenolic acid abundant in corn, wheat, and flax, exhibiting broad spectrum biological activities including anti-inflammatory [23], anti-diabetic [24], anticancer [25], and cardioprotective activity [26]. A survey of the literature reveals that ferulic acid contributes to the inhibition of the inflammatory responses in LPS-induced RAW 264.7 macrophages through inactivation of NF-κB pathway [27]. As typical examples, Nie and co-workers reported that ferulic acid could positively modulate the inflammatory response to septic liver injury, dose-dependently increase the viability of RAW264.7 cells and decrease the levels of pro-inflammatory factors [28]. Wu verified that ferulic acid can alleviate lipopolysaccharide-induced acute lung injury through the TLR4/NF-κB signaling pathway [29]. Despite the excellent anti-inflammatory activity displayed by ferulic acid and sesquiterpene lactones, their limited water solubility and bioavailability significantly restrict their clinical applications. A comprehensive investigation is necessary to determine whether synthesizing hybrid molecules, leveraging the synergistic effects of ferulic acid and sesquiterpene lactones, can enhance anti-inflammatory and anti-acute lung injury activities. Thus, we designed and synthesized a series of FA-PTL, FA-MCL hybrids, and evaluated their anti-inflammatory activity and the ability to ameliorate the acute lung injury induced by bleomycin (BLM) (Figure 1, compounds **1**–**6**). Furthermore, compound **6** was further tested as a novel anti-inflammatory agent for treatment of ALI in an animal model of ALI.

## 2. Results

### 2.1. Chemistry

**Scheme 1:**
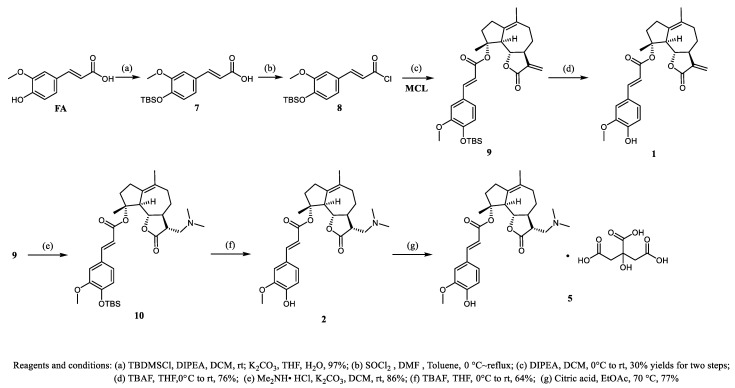
Synthesis of compounds **1** and **5.**

**Scheme 2:**
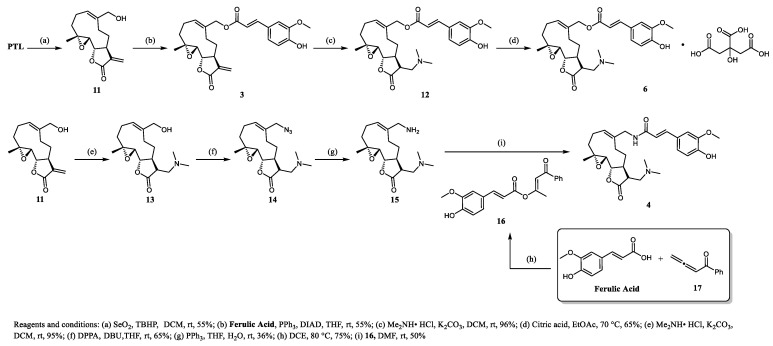
Synthesis of compounds **4** and **6.**

The preparation of ferulic acid-parthenolide (FA-PTL) or ferulic acid-micheliolide (FA-MCL) hybrids is illustrated in Scheme 1 and Scheme 2. As shown in Scheme 1, protection of the hydroxyl groups in ferulic acid with tert-butyldimethylchlorosilane (TBSCl) afforded compound **8** in 97% yield. Treatment of compound **8** with thionyl chloride gave crude corresponding acyl chloride intermediate, which reacted with MCL to afford compound **9** in 30% isolated yield for two steps. Then, compound **1** was obtained by the removal of the protecting group. Michael addition of compound **9** with dimethylamine hydrochloride at room temperature gave compound **10** in 86% yield. Deprotection of compound **10** gave compound **2** in moderate yield. Finally, the reaction of compound **2** with citric acid gave **5** in 77% yield.

As shown in Scheme 2, MMB (**11**) was obtained by the allylic oxidation of PTL in the presence of SeO_2_ and TBHP. The required compound **3** was prepared from MMB via Mitsunobu reaction. The compound **3** reacted with dimethylamine hydrochloride via Michael addition, followed by mixture with citric acid to obtain salt **6**. The Michael addition reaction between MMB (**11**) and dimethylamine hydrochloride afforded compound **13** in excellent yield. The resulting additive product **13** can be easily converted into azide **14,** followed by PPh_3_-promoted reduction of the azide functional group successfully gave compound **15**. Ferulic acid reacted smoothly with allenone **17** via 1,4-addition/isomerization cascade reaction to furnish compound **16** under mild conditions [30]. The reaction of compound **16** with amine **15** by aminolysis, gave compound **4** in a moderate yield.

### 2.2. Assessment of Cytotoxicity

CCK-8 Cell Proliferation was used to confirm the non-cytotoxic dose of those derivatives in RAW264.7 cells. However, IC_50_ values of those compounds show visible difference in vitro (Table 1). Considering the safety of compound **6** in RAW264.7 cells, a follow-up experiment was carried out at the concentration of 2 μM.

### 2.3. Anti-Inflammation of Compounds in RAW264.7 Cells

**Figure 2:**
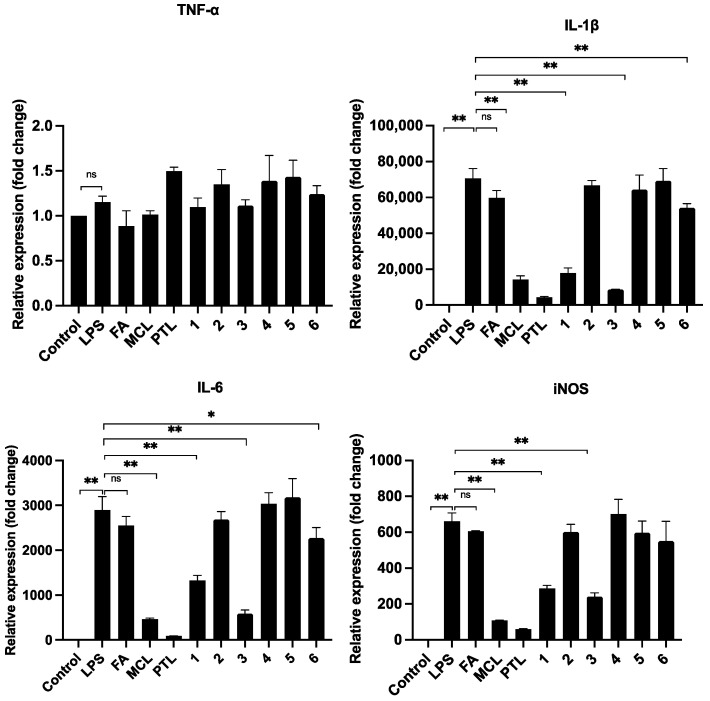
Anti-inflammation of 1–6 in RAW264.7 Cells. Expression of proinflammatory cytokines in RAW264.7 cells use RT-PCR. Cells (1 × 10^6^ cells/well) were seeded into 6-well plates and then pro-treated with **1**–**6** for 2 h. Cells were then challenged with LPS (1 μg/mL) or medium alone for 6 h and Total RNA was extracted from cells with Trizol reagent. TNF-α: tumor necrosis factor-α; IL-1β:Interleukin-1β; L-6: Interleukin-6. Data were analyzed by One-Way ANOVA. *: *p* < 0.05; **: *p* < 0.01. ns: no significance.

Inflammatory factors, including TNF-α, IL-1β, and IL-6, are involved in and accelerate inflammatory responses during inflammatory progression. In addition, iNOS (Nitric oxide synthase)-derived monocytes contribute to macrophage recruitment in the lung, and pulmonary-derived iNOS is detrimental during acute lung injury [31]. Moreover, observations indicate that inhibiting iNOS can reduce inflammation in the early stages of the bleomycin model of acute lung injury [32]. Therefore, the effective suppression of inflammation is considered an effective marker for the treatment of inflammation. To establish the efficacy of those compounds suppressing inflammatory procession, we tested pro-inflammatory cytokines that are released from the RAW264.7 cells upon LPS stimulation. Preliminary results showed that **1** and **3** could reduce the gene expression levels of IL-1β, IL-6 and iNOS significantly compared with LPS stimulation in macrophages (Figure 2). Noticeably, although FA-MCL hybrid **1** showed significant inhibitory in pro-inflammatory cytokines, its corresponding Michael adduct **2** showed decreased activity compared with compound **1**. Similarly, neither the amide derivative of FA-PTL hybrid **4** or **5** (the prodrug of **2** containing citric acid) showed anti-inflammation activities.

**Figure 3:**
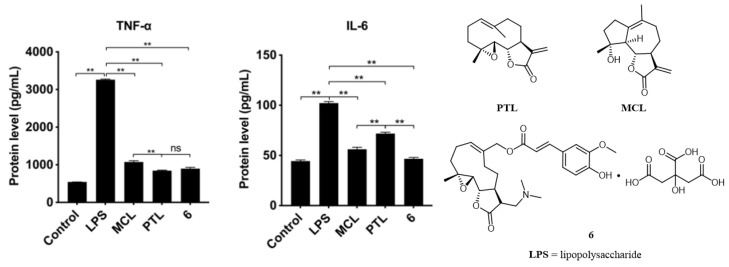
Protein levels of pro-inflammatory RAW264.7 Cells. RAW 264.7 cells were seeded into 12-well plates and then pro-treated with **6** for 6 h. Cells were then stimulated with LPS for 24 h. After 24 h incubation, the supernatant were collected and stored at −20 °C. The levels of TNF-α and IL-1β in the supernate were measured using the ELISA kit according to the manufacturer’s instructions. (**Left**,**middle**) pictures show the protein levels of TNF-α and IL-1β. (**Right**) picture shows the structure of compound **6**. Data were analyzed using One-Way ANOVA. **: *p* < 0.01. ns: no significance.

Although compounds **1**, **3**, and **6** demonstrate comparable activities, compound **6** stands out with outstanding water solubility, a feature not present in compounds **1** and **3** (Table 2). Compound **6**, the citrate salt of compound **12**, is obtained through the Michael reaction of compound **3** with dimethylamine. The use of Michael adducts can serve to modulate the release rate of active drugs, thereby enabling better control over the pharmacological effects of such compounds. For example, ACT001 is a salt that can dissolve in the stomach (pH = 1–3). Subsequently, upon entering the intestines, as the pH increases ACT001 converts to its prodrug form (DMAMCL), which is slowly absorbed. After absorption into the bloodstream (pH = 7.4), the active ingredient MCL is slowly released through a retro-Michael addition reaction. Through the combination of slow absorption and slow release, the blood concentration of the active ingredient can be controlled, thereby significantly increasing safety and oral bioavailability while maintaining therapeutic efficacy. ACT001 has demonstrated favorable therapeutic effects in various disease models, including brain tumors [19,33]. Similarly, compound **6** showed the potential for decreasing the expression of proinflammatory cytokines IL-1β, IL-6 and iNOS (Figure 1 and Figure 2). We next detected the protein levels of TNF-α and IL-6 by ELISA assay and found that **6** could also inhibit the release of pro-inflammatory cytokines in vitro (Figure 3). Given that compound **6** had better overall properties than **1** and **3**, it was chosen for further exploration.

### 2.4. Compound **6** Mitigated the Inflammation of BLM-Induced Mice

**Figure 4:**
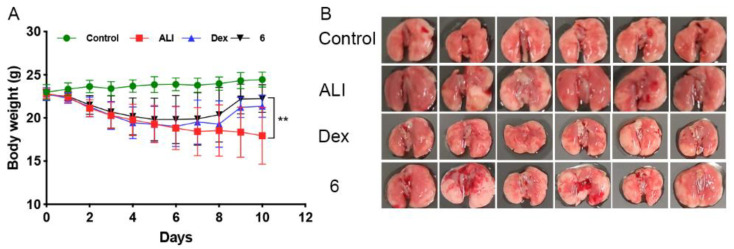
Effect of compound **6** on body weight and survival after bleomycin-induced acute lung injury in mice. After bleomycin (4 mg/kg, 25 μL), mice were treated (i.p) daily with compound **6** (10 mg/kg, 100 μL) for 14 days. Body weight variations ((**A**), *n* = 12) and percentage (%) of mouse survival ((**B**), *n* = 5–12) were compared between the four groups. (Values are means ± SEM, ** *p* < 0.01).

**Figure 5:**
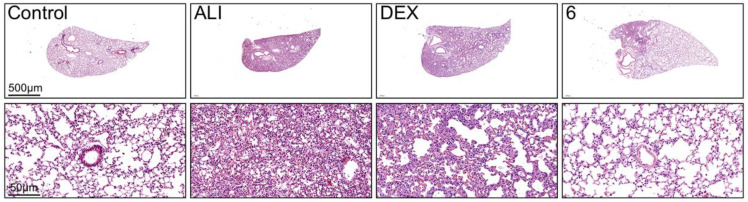
Pathological image of the protective effect of compound 6 on the ALI mice. Pathological image (×2, ×20) observation of mouse lung tissue sections stained with HE.

To further confirm the anti-inflammation effect of compound **6** in vivo, we made the acute-lung-injury model in C57/6j mice using bleomycin. Part weight gain was observed in the control group as a function of time, and lung delivery of bleomycin (BLM, 2.5 mg/kg) caused a significant weight loss. In contrast, daily (i.p) treatment with compound **6** (10 mg/kg) in mice with BLM-induced lung injury prevented the weight loss significantly and mitigated the acute lung injury induced by BLM (Figure 4A,B). However, treatment of **6** did not reverse the survival rate of BLM mice. Moreover, the effect of dexamethasone and the compound **6** was not observed during the acute exudative phase (i.e., at 3–7 days) as we expected. To further investigate the protective effect of **6** by BLM-induced ALI, the pathological changes in lung tissues were studied (Figure 5). The results of lung H&E staining showed that there were a large number of inflammatory cell infiltrates in the lung tissues of mice in the BLM group, accompanied by edema and destruction of the alveolar structure. Compound **6** reduced inflammatory cell infiltration and edema in lung tissue of mice, and improved alveolar structure. These results indicated that compound **6** significantly inhibited cell infiltration in lung tissue and had a good protective effect on BLM-induced acute lung injury in mice.

### 2.5. Transformation from Prodrug to Active Drug

**Figure 6:**
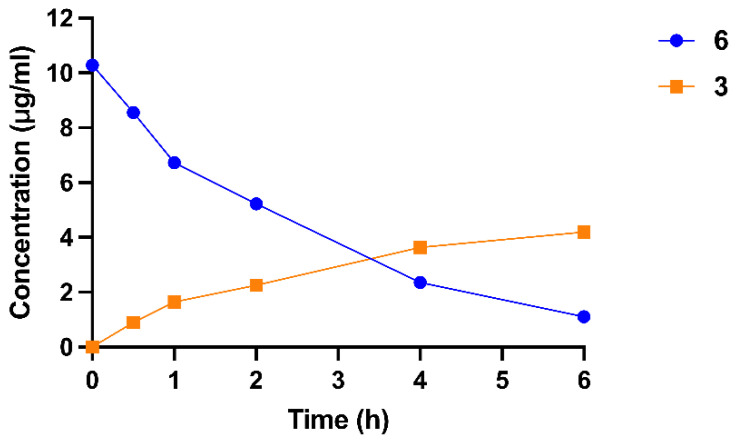
The stability of compound **6** in pH7.4 HEPES buffer.

The Michael addition reaction has emerged as an effective synthetic strategy to improve the water solubility and pharmaceutical efficacy of active drug compounds [34]. For example, DMAPT is an adduct of parthenolide that exhibits a solubility more than 1000 times greater than its parent compound and has entered clinical trials in the United Kingdom for the treatment of various hematological malignancies, including acute myeloid leukemia (AML), acute lymphoblastic leukemia (ALL), and chronic lymphocytic leukemia (CLL) [35]. In addition, the use of Michael addition products can also serve to modulate the release rate of active drugs, thereby enabling better control over the pharmacological effects of such compounds. Thus, the experiment of converting the prodrug compound (**6**) into compound (**3**) was evaluated in a HEPES buffer solution with a pH of 7.4. Compound **6** rapidly releases compound **3** under these conditions. (Figure 6, T_1/2_ = 2 h). Aqueous solubility of compounds **1**–**6** was also investigated, the results show that compounds **1**–**4** possessed worse solubility than parthenolide and micheliolide at pH 7.0. However, salts **5** (Sol. = µg/mL, pH = 7.0) and **6** (Sol. = µg/mL, pH = 7.0) showed significantly improved water solubility at pH 7.0, which was much greater than that of parthenolide and micheliolide.

## 3. Experimental Procedure and Methods

### 3.1. Chemistry

All reagents and solvent were commercially available at analytical grade and were used as received. The used solvents were purified and dried according to common procedures. High-resolution mass spectra (HRMS) were obtained with a FTICR-MS (Ionspec 7.0T) spectrometer (Lebrilla League, Davis, CA, USA). The ^1^H and ^13^C NMR spectra were recorded on a Bruker AV 400 MHz spectrometer (Billerica, MA, USA) and calibrated by using internal references and solvent signals CDCl_3_ (δ_H_ 7.26, δ_C_ 77.00), D_2_O (δ_H_ 4.80) and DMSO-*d*_6_ (δ_H_ 2.50, δ_C_ 40) solution. The following abbreviations were used to explain multiplicities: s = singlet, d = doublet, t = triplet, q = quartet, m = multiplet, br = broad). Flash column chromatography was performed over silica gel 200−300 mesh, and the eluent was a mixture of ethyl acetate (EA) and petroleum ether (PE) or a mixture of dichloromethane (DCM) and Methanol (MeOH). High-resolution mass spectra (HRMS) were detected by Q Exactive Focus LC-MS (Thermo, Waltham, MA, USA). HPLC data were recorded on Shimadzu LC-20AT (Tokyo, Japan). For the ^1^H and ^13^C NMR spectra of compounds (**7**, **9**, **1**, **10**, **2**, **5**, **3**, **12**, **6**, **13**, **14**, **15**, **16**, and **4**), please see the supporting information (Figures S1–S26).

#### 3.1.1. Compound **9**

*(3aS,9R,9bS)-6,9-dimethyl-3-methylene-2-oxo-2,3,3a,4,5,7,8,9,9a,9b-decahydroazuleno[4,5-b]furan-9-yl (E)-3-(4-((tert-butyldimethylsilyl)oxy)-3-methoxyphenyl)acrylate* (**9**). To a solution of compound **7** (5 mmol) in Toluene (2 mL) was added SOCl_2_ (2 mL) and DMF (a drop) at 0 °C under N_2_ atmosphere. Then the mixture was stirred at 80 °C for 1 h. Removal of the organic layer under vacuum gave crude acyl chloride.

To a solution of MCL (2.5 mmol) in anhydrous CH_2_Cl_2_ (5 mL) was added DIPEA (5 mmol) at 0 °C under N_2_ atmosphere. Then the crude acyl chloride in anhydrous CH_2_Cl_2_ (2 mL) was added dropwise at this temperature. The mixture was stirred at room temperature for 6h. The reaction was quenched with saturated aqueous NH_4_Cl and extracted with CH_2_Cl_2_ (3 × 15 mL). The combined organic layers were washed with saturated brine, dried over Na_2_SO_4_, and concentrated to give an oily crude product, which was purified on a silica gel column (hexanes: EtOAc = 10:1) to yield compound **9** as a colorless oil. Yield: 30%; ^1^H NMR (400 MHz, CDCl_3_): *δ* = 7.66 (d, *J* = 15.9 Hz, 1H), 7.08–7.03 (m, 2H), 6.82 (d, *J* = 8.1 Hz, 1H), 6.27–6.20 (m, 2H), 5.48 (d, *J* = 3.1 Hz, 1H), 3.88–3.83 (m, 4H), 3.15 (d, *J* = 10.1 Hz, 1H), 2.75–2.70 (m, 1H), 2.63–2.57 (m, 1H), 2.51–2.46 (m, 1H), 2.28 (s, 3H), 2.23–2.09 (m, 1H), 2.04–1.95 (m, 1H), 1.73 (s, 3H), 1.62 (s, 1H), 1.59 (s, 3H), 0.98 (s, 9H), 0.16 (s, 6H). ^13^C NMR (100 MHz, CDCl_3_): *δ* = 170.4, 166.6, 151.1, 147.1, 144.7, 139.4, 131.6, 129.9, 128.6, 122.3, 120.9, 118.8, 117.4, 110.8, 88.4, 83.1, 57.3, 55.4, 49.9, 36.6, 34.9, 30.6, 25.9, 25.6, 24.2, 18.5, 18.4, −4.7. HRMS (ESI): *m*/*z* [M + H]^+^ calcd for C_31_H_43_O_6_Si: 539.2823; found: 539.2821.

#### 3.1.2. Compound **1**

*(3aS,9R,9bS)-6,9-dimethyl-3-methylene-2-oxo-2,3,3a,4,5,7,8,9,9a,9b-decahydroazuleno[4,5-b]furan-9-yl (E)-3-(4-hydroxy-3-methoxyphenyl)acrylate* (**1**). To a solution of compound **9** (0.9 mmol) in THF (3 mL) was added TBAF (1.8 mmol) at 0 °C. Then the reaction mixture was stirred at room temperature for 40 min. The reaction was quenched with saturated ammonium chloride solution and extracted with EtOAc (3 × 15 mL). The combined organic layers were washed with saturated brine, dried over Na_2_SO_4_, and concentrated to give an oily crude product, which was purified on a silica gel column (hexanes: EtOAc = 3:1) to yield compound **1** as a yellow oil. Yield: 76%; ^1^H NMR (400 MHz, CDCl_3_): δ = 7.66 (d, *J* = 15.9 Hz, 1H), 7.09–7.07 (m, 2H), 6.89 (d, *J* = 8.7 Hz, 1H), 6.27–6.21 (m, 2H), 5.49 (d, *J* = 3.1 Hz, 1H), 3.92 (s, 3H), 3.86 (t, *J* = 10.1 Hz, 1H), 3.15 (d, *J* = 10.2 Hz, 1H), 2.75–2.70 (m, 1H), 2.63–2.58 (m, 1H), 2.51–2.45 (m, 1H), 2.28 (s, 3H), 2.12–2.09 (m, 1H), 2.04–1.95 (m, 1H), 1.72 (d, *J* = 1.8 Hz, 3H), 1.59 (s, 3H), 1.43–1.33 (m, 1H), 1.28–1.21 (m, 1H).^13^C NMR (100 MHz, CDCl_3_): δ = 170.4, 166.6, 147.7, 146.7, 144.7, 139.5, 131.6, 129.9, 127.3, 123.1, 118.8, 117.0, 114.6, 109.6, 88.5, 83.1, 57.3, 55.9, 49.9, 36.7, 35.0, 30.6, 25.9, 24.2, 18.5. HRMS (ESI): *m*/*z* [M + H]^+^ calcd for C_25_H_29_O_6_: 425.1959; found: 425.1957.

#### 3.1.3. Compound **10**

*(3R,3aS,9R,9bS)-3-((dimethylamino)methyl)-6,9-dimethyl-2-oxo-2,3,3a,4,5,7,8,9,9a,9b-decahydroazuleno[4,5-b]furan-9-yl (E)-3-(4-((tert-butyldiphenylsilyl)oxy)-3-methoxyphenyl)acrylate* (**10**). Compound **9** (0.6 mmol), K_2_CO_3_ (9 mmol) and Me_2_NH·HCl (4.8 mmol) were added to dry DCM (5 mL) at room temperature and the resulting solution was stirred at this temperature for 5 h. The solid in the mixture was filtered off, and the resulting solution was concentrated under reduced pressure. The residue was dissolved in CH_2_Cl_2_, and then washed with water. The organic layer was dried with Na_2_SO_4_ and concentrated under reduced pressure. The residue was purified by column chromatography on silica gel using ethyl acetate−petroleum ether as the eluent to give the desired product **10** as a yellow oil. Yield: 86%; ^1^H NMR (400 MHz, CDCl_3_):δ = 7.66 (d, *J* = 15.9 Hz, 1H), 7.09–7.01 (m, 2H), 6.81 (d, *J* = 8.1 Hz, 1H), 6.24 (d, *J* = 15.9 Hz, 1H), 3.87–3.80 (m, 4H), 3.07 (d, *J* = 9.0 Hz, 1H), 2.74 (dd, *J* = 12.9, 4.8 Hz, 1H), 2.64–2.54 (m, 2H), 2.50–2.35 (m, 2H), 2.26 (s, 6H), 2.19–1.89 (m, 5H), 1.70 (s, 3H), 1.58 (s, 3H), 1.37–1.13 (m, 2H), 0.98 (s, 9H), 0.16 (s, 6H).^13^C NMR (100 MHz, CDCl_3_): δ = 177.5, 166.6, 151.1, 147.1, 144.7, 131.9, 129.9, 128.7, 122.1, 120.9, 117.5, 111.1, 88.6, 82.5, 58.5, 56.9, 55.4, 51.5, 46.0, 45.9, 44.94, 36.8, 35.4, 30.4, 27.4, 25.7, 23.9, 18.6, 18.5, −4.6. HRMS (ESI): *m*/*z* [M + H]^+^ calcd for chemical formula: C_33_H_50_NO_6_Si: 584.3402; found: 584.3399.

#### 3.1.4. Compound **2**

*(3R,3aS,9R,9aS,9bS)-3-((dimethylamino)methyl)-6,9-dimethyl-2-oxo-2,3,3a,4,5,7,8,9,9a,9b-decahydroazuleno[4,5-b]furan-9-yl (E)-3-(4-hydroxy-3-methoxyphenyl)acrylate* (**2**). To a solution of compound **10** (0.6 mmol) in THF (3 mL) was added TBAF at 0 °C. Then the reaction mixture was stirred at room temperature for 40 min. The reaction was quenched with saturated ammonium chloride solution and extracted with EtOAc (3 × 15 mL). The combined organic layers were washed with saturated brine, dried over Na_2_SO_4_, and concentrated to give an oily crude product, which was purified on a silica gel column (CH_2_Cl_2_: MeOH = 20:1) to yield compound **2** as a yellow solid. Yield: 64%. mp: 139−141 °C. ^1^H NMR (400 MHz, CDCl_3_):δ = 7.64 (d, *J* = 15.9 Hz, 1H), 7.06 (d, *J* = 8.6 Hz, 2H), 6.86 (d, *J* = 7.7 Hz, 1H), 6.22 (d, *J* = 15.9 Hz, 1H), 3.90 (s, 3H), 3.84 (t, *J* = 10.1 Hz, 1H), 3.05 (d, *J* = 10.0 Hz, 1H), 2.74 (dd, *J* = 13.0, 4.9 Hz, 1H), 2.67–2.53 (m, 2H), 2.50–2.35 (m, 2H), 2.27–1.96 (m, 12H), 1.69 (s, 3H), 1.57 (s, 3H), 1.38–1.19 (m, 2H). ^13^C NMR (100 MHz, CDCl_3_): δ = 177.5, 166.7, 147.8, 146.8, 144.7, 131.9, 129.8, 127.1, 122.9, 116.9, 114.7, 109.8, 88.6, 82.5, 58.2, 56.9, 55.9, 51.4, 45.9, 44.9, 36.7, 35.3, 30.4, 27.3, 23.9, 18.5. HRMS (ESI): *m*/*z* [M + H]^+^ calcd for chemical formula: C_27_H_36_NO_6_: 470.2537; found: 470.2538.

#### 3.1.5. Compound **5**

*(3R,3aS,9R,9aS,9bS)-3-((dimethylamino)methyl)-6,9-dimethyl-2-oxo-2,3,3a,4,5,7,8,9,9a,9b-decahydroazuleno[4,5-b]furan-9-yl (E)-3-(4-hydroxy-3-methoxyphenyl)acrylate 2-hydroxypropane-1,2,3-tricarboxylate* (**5**). To a solution of compound **2** (1.0 mmol) in EtOAc (10 mL) was added citric acid (1.0 mmol) at room temperature. Then the reaction mixture was stirred at room temperature for 30 min. The white solid in the mixture was filtered off, and washed with EtOAc to obtain compound **5**. Yield: 77%. mp: 96−98 °C. ^1^H NMR (400 MHz, DMSO-d6): 9.58 (s, 1H), 7.57 (d, *J* = 15.8 Hz, 1H), 7.28 (d, *J* = 1.8 Hz, 1H), 7.08 (dd, *J* = 8.2, 1.8 Hz, 1H), 6.78 (d, *J* = 8.1 Hz, 1H), 6.31 (d, *J* = 15.8 Hz, 1H), 4.0–3.95 (m, 1H), 3.81 (s, 3H), 3.07 (d, *J* = 9.8 Hz, 1H), 2.83–2.76 (m, 3H), 2.65–2.49 (m, 4H), 2.48–2.28 (m, 8H), 2.27–2.00 (m, 5H), 1.95–1.87 (m, 1H), 1.68 (s, 3H), 1.49 (s, 3H), 1.36–1.29 (m, 1H). ^13^C NMR (100 MHz, DMSO-d6): δ = 177.5, 176.9, 170.8, 166.4, 149.7, 148.4, 145.4, 132.1, 130.1, 126.2, 123.5, 116.4, 115.9, 111.6, 88.7, 82.2, 71.9, 57.4, 56.3, 56.1, 50.9, 45.4, 44.4, 43.6, 36.8, 35.2, 30.3, 26.8, 24.3, 21.2, 14.6. HRMS (ESI): *m*/*z* [M + H]^+^ calcd for chemical formula: C_27_H_36_NO_13_: 470.2537; found: 470.2539.

#### 3.1.6. Compound **3**

*((1aR,7aS,10aS,10bS,E)-1a-methyl-8-methylene-9-oxo-1a,2,3,6,7,7a,8,9,10a,10b-decahydrooxireno[2′,3′:9,10]cyclodeca[1,2-b]furan-5-yl)methyl (E)-3-(4-hydroxy-3-methoxyphenyl)acrylate* (**3**). To a solution of MMB (5 mmol), Ferulic acid (7.5 mmol) and PPh_3_ (7.5 mmol) in anhydrous THF (50 mL) was added DIAD (5 mmol) at room temperature under N_2_ atmosphere. The reaction was stirred for 4h at room temperature. The reaction was quenched with saturated ammonium chloride solution and extracted with EtOAc (3 × 30 mL). The combined organic layers were washed with saturated brine, dried over Na_2_SO_4_, and concentrated. The crude product was purified on a silica gel column (PE: EA = 2:1) to yield compound **3** as a yellow solid. Yield: 55%; mp: 96−98 °C. ^1^H NMR (400 MHz, CDCl_3_) δ = 7.60 (d, *J* = 15.9 Hz, 1H), 7.05 (dd, *J* = 8.2, 1.8 Hz, 1H), 6.99 (d, *J* = 1.8 Hz, 1H), 6.91 (d, *J* = 8.2 Hz, 1H), 6.24 (dd, *J* = 9.7, 6.2 Hz, 2H), 6.03 (s, 1H), 5.73 (t, *J* = 8.1 Hz, 1H), 5.55 (d, *J* = 3.2 Hz, 1H), 4.76 (d, *J* = 12.5 Hz, 1H), 4.59 (d, *J* = 12.5 Hz, 1H), 3.92 (s, 3H), 3.87 (t, *J* = 9.3 Hz, 1H), 3.01 (m, *J* = 9.1, 4.7 Hz, 1H), 2.90 (d, *J* = 9.4 Hz, 1H), 2.47–2.16 (m, 6H), 1.72–1.65 (m, 1H), 1.55 (s, 3H), 1.12 (t, *J* = 12.6 Hz, 1H).

#### 3.1.7. Compound **12**

*((1aR,7aS,8R,10aS,10bS,E)-8-((dimethylamino)methyl)-1a-methyl-9-oxo-1a,2,3,6,7,7a,8,9,10a,10b-decahydrooxireno[2′,3′:9,10]cyclodeca[1,2-b]furan-5-yl)methyl (E)-3-(4-hydroxy-3-methoxyphenyl)acrylate* (**12**). Compound **3** (1 mmol), K_2_CO_3_ (15 mmol) and Me_2_NH·HCl (8 mmol) were added to dry DCM (10 mL) at room temperature and the resulting solution was stirred at this temperature for 5 h. The solid in the mixture was filtered off, and the resulting solution was concentrated under reduced pressure. The residue was dissolved in CH_2_Cl_2_, and then washed with water. The organic layer was dried with Na_2_SO_4_ and concentrated under reduced pressure. The residue was purified by column chromatography on silica gel using ethyl acetate−petroleum ether as the eluent to give the desired product **12** as a yellow oil. Yield: 96%; ^1^H NMR (400 MHz, CDCl_3_): δ = 7.62 (d, *J* = 15.9 Hz, 1H), 7.08 (dd, *J* = 8.2, 1.8 Hz, 1H), 7.03–7.02 (m, 1H), 6.92 (d, *J* = 8.2 Hz, 1H), 6.30 (d, *J* = 15.9 Hz, 1H), 5.66 (t, *J* = 8.0 Hz, 1H), 4.87 (d, *J* = 12.8 Hz, 1H), 4.66 (d, *J* = 12.9 Hz, 1H), 3.92 (s, 3H), 3.90–3.84 (m, 2H), 2.81 (d, *J* = 9.4 Hz, 1H), 2.78–2.74 (m, 1H), 2.64 (dd, *J* = 12.9, 5.7 Hz, 1H), 2.51–2.28 (m, 6H), 2.24 (s, 6H), 2.18–2.12 (m, 2H), 1.59–1.54 (m, 4H), 1.10 (t, *J* = 12.7 Hz, 1H). ^13^C NMR (100 MHz, CDCl_3_) δ = 176.9, 166.8, 148.1, 146.8, 145.2, 135.9, 128.5, 126.8, 123.1, 114.9, 114.7, 109.3, 81.2, 66.2, 63.8, 59.8, 58.2, 55.9, 45.7, 44.4, 42.9, 36.9, 26.9, 24.5, 23.7, 17.9. HRMS (ESI): *m*/*z* [M + H]^+^ calcd for chemical formula: C_27_H_36_NO_7_: 486.2486; found: 486.2485.

#### 3.1.8. Compound **6**

*((1aR,7aS,8R,10aS,10bS,E)-8-((dimethylamino)methyl)-1a-methyl-9-oxo-1a,2,3,6,7,7a,8,9,10a,10b-decahydrooxireno[2′,3′:9,10]cyclodeca[1,2-b]furan-5-yl)methyl (E)-3-(4-hydroxy-3-methoxyphenyl)acrylate 2-hydroxypropane-1,2,3-tricarboxylate* (**6**). To a solution of compound **12** (1 mmol) in EtOAc (10 mL) was added citric acid (1 mmol) at room temperature. Then the reaction mixture was stirred at 70 °C for 8 h. The white solid in the mixture was filtered off, and washed with EtOAc to obtain compound **6**. Yield: 65%. ^1^H NMR (400 MHz, D_2_O): δ = 7.49 (d, *J* = 15.9 Hz, 1H), 7.08 (d, *J* = 1.7 Hz, 1H), 7.01 (dd, *J* = 8.2, 1.6 Hz, 1H), 6.82 (d, *J* = 8.2 Hz, 1H), 6.27 (d, *J* = 16.0 Hz, 1H), 5.56 (t, *J* = 8.0 Hz, 1H), 4.59 (d, *J* = 13.1 Hz, 1H), 4.43 (d, *J* = 13.0 Hz, 1H), 4.21 (t, *J* = 9.6 Hz, 1H), 3.78 (s, 3H), 3.45–3.39 (m, 1H), 3.28 (dd, *J* = 13.2, 3.5 Hz, 1H), 3.16–3.06 (m, 1H), 2.92–2.84 (m, 7H), 2.79–2.63 (m, 4H), 2.38–2.12 (m, 4H), 2.09–1.95 (m, 4H), 1.76–1.70 (m, 1H), 1.49 (s, 3H), 1.16 (t, *J* = 7.2 Hz, 1H), 0.93 (t, *J* = 12.2 Hz, 1H). ^13^C NMR (100 MHz, D_2_O): δ = 177.8, 177.4, 174.0, 168.7, 148.2, 147.7, 146.2, 134.4, 129.5, 126.5, 123.2, 115.6, 114.2, 111.2, 82.2, 73.5, 66.6, 63.4, 62.7, 61.7, 55.8, 55.5, 43.4, 42.4, 41.3, 35.7, 24.9, 24.0, 22.9, 20.5, 16.6, 13.2. HRMS (ESI): *m*/*z* [M + H]^+^ calcd for Chemical Formula: C_27_H_36_NO_7_: 486.2486; found: 486.2481.

#### 3.1.9. Compound **13**

*(1aR,7aS,8R,10aS,10bS,E)-8-((dimethylamino)methyl)-5-(hydroxymethyl)-1a-methyl-2,3,6,7,7a,8,10a,10b-octahydrooxireno[2′,3′:9,10]cyclodeca[1,2-b]furan-9(1aH)-one* (**13**). MMB (3 mmol), K_2_CO_3_ (45 mmol) and Me_2_NH·HCl (24 mmol) were added to dry DCM (25 mL) at room temperature and the resulting solution was stirred at this temperature for 5 h. The solid in the mixture was filtered off, and the resulting solution was concentrated under reduced pressure. The residue was dissolved in CH_2_Cl_2_, and then washed with water. The organic layer was dried with Na_2_SO_4_ and concentrated under reduced pressure. The residue was purified by column chromatography on silica gel using ethyl acetate−petroleum ether as the eluent to give the desired product **13** as a yellow oil. Yield: 95%; ^1^H NMR (400 MHz, CDCl_3_): δ = 5.57 (t, *J* = 8.0 Hz, 1H), 4.09 (dd, *J* = 31.7, 13.1 Hz, 2H), 3.84 (t, *J* = 9.0 Hz, 1H), 2.81–2.78 (m, 1H), 2.74–2.68 (m, 1H), 2.65–2.55 (m, 1H), 2.49–2.23 (m, 6H), 2.23–2.20 (m, 7H), 2.14–2.07 (m, 2H), 1.62–1.54 (m, 1H), 1.51 (s, 3H), 1.06 (t, *J* = 12.8 Hz, 1H). ^13^C NMR (100 MHz, CDCl_3_): δ = 176.9, 140.8, 127.0, 81.4, 65.8, 63.9, 59.9, 57.5, 45.6, 44.0, 42.0, 36.9, 27.3, 25.6, 23.6, 17.8.

#### 3.1.10. Compound **14**

*(1aR,7aS,8R,10aS,10bS,E)-5-(azidomethyl)-8-((dimethylamino)methyl)-1a-methyl-2,3,6,7,7a,8,10a,10b-octahydrooxireno[2′,3′:9,10]cyclodeca[1,2-b]furan-9(1aH)-one* (**14**). To a solution of compound **13** (1 mmol) and DBU (2 mmol) in THF (10 mL) was added DPPA at room temperature. The reaction mixture was stirred at room temperature for 8h. Removal of the organic layer under vacuum gave a yellow oil. Then the crude product was added to EtOAc (10 mL)/H_2_O (10 mL) at room temperature. The reaction was extracted with EtOAc (3 × 30 mL). The combined organic layers were washed with saturated brine, dried over Na_2_SO_4_, and concentrated. The crude product was purified on a silica gel column (PE: EA = 1:1) to yield compound **14** as a colorless oil. Yield: 65%. ^1^H NMR (400 MHz, CDCl_3_): δ = 5.60 (t, *J* = 8.1 Hz, 1H), 3.95 (d, *J* = 13.2 Hz, 1H), 3.87–3.83 (m, 2H), 2.76–2.56 (m, 2H), 2.61–2.56 (m, 1H), 2.47–2.35 (m, 3H), 2.34–2.08 (m, 11H), 1.64–1.56 (m, 1H), 1.54 (s, 3H), 1.07 (t, *J* = 12.6 Hz, 1H).^13^C NMR (100 MHz, CDCl_3_): δ = 176.8, 135.7, 129.6, 81.0, 63.9, 59.8, 58.1, 55.4, 45.6, 44.1, 42.6, 36.9, 26.6, 24.4, 23.7, 17.8. HRMS (ESI): *m*/*z* [M + H]^+^ calcd for chemical formula: C_17_H_27_N_4_O_3_: 335.2083; found: 335.2085.

#### 3.1.11. Compound **15**

*(1aR,7aS,8R,10aS,10bS,E)-5-(aminomethyl)-8-((dimethylamino)methyl)-1a-methyl-2,3,6,7,7a,8,10a,10b-octahydrooxireno[2′,3′:9,10]cyclodeca[1,2-b]furan-9(1aH)-one* (**15**). To a solution of compound **14** (0.4 mmol) in THF (3 mL) was added PPh_3_ (0.44 mmol) and H_2_O (45 µL). The reaction mixture was stirred at room temperature overnight. Removal of the organic layer under vacuum gave a yellow oil. The crude product was purified on a silica gel column (DCM: CH_2_Cl_2_ = 6:1) to yield compound **15** as a colorless oil. Yield: 36%. ^1^H NMR (400 MHz, CDCl_3_): δ = 5.50 (t, *J* = 7.9 Hz, 1H), 3.82 (d, *J* = 9.5 Hz, 1H), 3.45 (s, 1H), 3.33 (d, *J* = 6.3 Hz, 1H), 2.80–2.71 (m, 2H), 2.57 (dd, *J* = 12.9, 6.5 Hz, 1H), 2.49 (s, 2H), 2.44–2.34 (m, 3H), 2.28–2.25 (m, 3H), 2.22 (s, 6H), 2.18–2.08 (m, 3H), 1.53 (s, 3H), 1.06 (t, *J* = 12.3 Hz, 1H).^13^C NMR (100 MHz, CDCl_3_): δ = 176.9, 141.2, 124.8, 81.2, 63.7, 60.0, 58.4, 45.7, 45.4, 44.3, 43.2, 37.1, 26.8, 24.6, 23.7, 17.9. HRMS (ESI): *m*/*z* [M + H]^+^ calcd for chemical formula: C_17_H_29_N_2_O_3_: 309.2173; found: 309.2174.

#### 3.1.12. Compound **16**

*(E)-4-oxo-4-phenylbut-2-en-2-yl (E)-3-(4-hydroxy-3-methoxyphenyl)acrylate* (**16**). To a solution of ferulic acid (1.1 mmol) in DCE (5 mL) was added allenone **17** (1 mmol) at room temperature. The reaction mixture was allowed to stir at 80 °C until the allenone **17** was fully consumed. The reaction mixture was purified by flash silica gel chromatography (PE: EA = 5:1) to afford the compound **16** as a brown oil. Yield: 75%. ^1^H NMR (400 MHz, CDCl_3_): *δ* = 7.95–7.92 (m, 2H), 7.74 (d, *J* = 15.9 Hz, 1H), 7.57–7.50 (m, 1H), 7.47–7.43 (m, 2H), 7.13–7.10 (m, 1H), 7.06 (d, *J* = 1.6 Hz, 1H), 6.95–6.90 (m, 2H), 6.36 (d, *J* = 15.9 Hz, 1H), 6.17–6.09 (m, 1H), 3.92 (s, 3H), 2.48 (d, *J* = 0.8 Hz, 3H); ^13^C NMR (100 MHz, CDCl_3_): *δ* = 190.4, 164.5, 164.2, 148.6, 147.3, 146.9, 138.7, 132.7, 128.5, 128.1, 126.4, 123.5, 114.9, 113.9, 113.3, 109.6, 55.9, 19.1.; HRMS (ESI): *m*/*z* [M + H]^+^ calcd for chemical formula: C_20_H_18_NaO_5_: 361.1046; found: 361.1045.

#### 3.1.13. Compound **4**

*(E)-N-(((1aR,7aS,8R,10aS,10bS,E)-8-((dimethylamino)methyl)-1a-methyl-9-oxo-1a,2,3,6,7,7a,8,9,10a,10b-decahydrooxireno[2′,3′:9,10]cyclodeca[1,2-b]furan-5-yl)methyl)-3-(4-hydroxy-3-methoxyphenyl)acrylamide* (**4**). To a solution of compound **15** (2 mmol) in dry DMF (10 mL) was added compound **16** at room temperature. The reaction mixture was allowed to stir at room temperature until the compound **16** was fully consumed. The reaction was quenched with brine and extracted with EtOAc (3 × 15 mL). The combined organic layers were washed with saturated brine, dried over Na_2_SO_4_, and concentrated. The crude product was purified on a silica gel column (DCM: CH_2_Cl_2_ = 15:1) to yield compound **4** as a yellow solid. mp: 132−134 °C. Yield: 50%. ^1^H NMR (400 MHz, DMSO-d6): δ = 9.40 (s, 1H), 8.00 (t, *J* = 5.8 Hz, 1H), 7.34 (d, *J* = 15.7 Hz, 1H), 7.13 (d, *J* = 1.8 Hz, 1H), 6.99 (dd, *J* = 8.2, 1.8 Hz, 1H), 6.78 (d, *J* = 8.1 Hz, 1H), 6.49 (d, *J* = 15.7 Hz, 1H), 5.41–5.29 (m, 1H), 4.01 (t, *J* = 9.5 Hz, 1H), 3.90–3.84 (m, 1H), 3.80 (s, 3H), 2.72 (d, *J* = 9.5 Hz, 1H), 2.64–2.55 (m, 3H), 2.47–2.19 (m, 5H), 2.17 (s, 6H), 2.08–2.00 (m, 3H), 1.64–1.54 (m, 1H), 1.47 (s, 3H), 0.94–0.84 (m, 1H). ^13^C NMR (100 MHz, DMSO-d6): δ = 177.8, 165.7, 148.7, 148.3, 139.6, 138.3, 126.9, 124.1, 121.9, 119.3, 116.1, 111.2, 81.1, 63.7, 60.2, 58.7, 55.9, 45.9, 43.9, 42.8, 42.3, 37.4, 26.2, 24.9, 23.6, 17.9. HRMS (ESI): *m*/*z* [M + H]+ calcd for chemical formula: C_27_H_37_N_2_O_6_: 485.2646; found: 485.2641.

#### 3.1.14. Compound **18**

*(E)-N-(((1aR,7aS,8R,10aS,10bS,E)-8-((dimethylamino)methyl)-1a-methyl-9-oxo-1a,2,3,6,7,7a,8,9,10a,10b-decahydrooxireno[2′,3′:9,10]cyclodeca[1,2-b]furan-5-yl)methyl)-3-(4-hydroxy-3-methoxyphenyl)acrylamide 2-hydroxypropane-1,2,3-tricarboxylate* (**18**). To a solution of compound **4** (2.0 mmol) in EtOAc (20 mL) was added citric acid (2.0 mmol) at room temperature. Then the reaction mixture was stirred at room temperature for 30 min. The white solid in the mixture was filtered off, and washed with EtOAc to obtain compound **18**. Yield: 70%. mp: 113−115 °C. ^1^H NMR (400 MHz, DMSO-d_6_): 9.42 (s, 1H), 8.02 (t, *J* = 5.7 Hz, 1H), 7.35 (d, *J* = 15.7 Hz, 1H), 7.13 (d, *J* = 1.8 Hz, 1H), 6.99 (dd, *J* = 8.2, 1.8 Hz, 1H), 6.79 (d, *J* = 8.1 Hz, 1H), 6.49 (d, *J* = 15.7 Hz, 1H), 5.36 (t, *J* = 7.4 Hz, 1H), 4.06–3.98 (m, 2H), 3.93–3.88 (m, 1H), 3.80 (s, 3H), 3.78–3.73 (m, 1H), 2.79–2.62 (m, 4H), 2.62–2.54 (m, 1H), 2.51–2.49 (m, 2H), 2.47–2.32 (m, 3H), 2.28 (s, 6H), 2.25–2.21 (m, 1H), 2.05–2.02 (m, 3H), 1.66–1.55 (m, 1H), 1.47 (s, 3H), 0.95–0.82 (m, 1H). ^13^C NMR (100 MHz, DMSO-d_6_): δ = 177.63, 177.08, 170.82, 165.79, 148.74, 148.28, 139.68, 138.27, 126.88, 124.42, 122.02, 119.28, 116.13, 111.22, 81.21, 71.83, 63.56, 60.23, 58.22, 55.98, 45.59, 44.58, 43.74, 42.78, 42.41, 37.37, 26.13, 24.75, 23.65, 21.24, 17.98, 14.57. HRMS (ESI): *m*/*z* [M + H]^+^ calcd for chemical formula: C_27_H_37_N_2_O_6_: 485.2646; found: 485.2643.

### 3.2. Cell Culture and Treatment

Mouse RAW264.7 macrophage were obtained from National Collection of Authenticated Cell Cultures (Beijing, China) and were maintained at 37 °C and in a 5% CO_2_ atmosphere in Dulbecco’s modified Eagle’s medium (DMEM), added with 10% fetal bovine serum (FBS) and 1% streptomycin–penicillin. Cell passage was limited in number to 15 times to avoid cell differentiation.

### 3.3. Cell Viability Assay

The toxicity of those compounds was determined by the CCK-8 assay. RAW264.7 cells were seeded in a 96-well plate at a density of 2 × 10^5^/mL and a volume of 100 μL/well. After incubation for 12 h at 37 °C, the cells were then incubated with compounds at concentrations of 1.25, 2.5, 5, 10, 20, 40 and 80 μM for 24 h, followed by the addition of 10 μL CCK-8 solution to each well, and the plates were further incubated for 2 h at 37 °C. The absorbance at a wavelength of 450 nm was measured using a microplate reader (Bio-Rad Laboratories, Inc., Hercules, CA, USA).

### 3.4. Isolation of Total RNA and Quantitative Real-Time Polymerase Chain Reaction (qRT-PCR)

Total RNA was isolated using TRIzol reagent (Takara, Ōtsu-shi, Japan) and then transcribed into cDNA using a transcriptor cDNA synthesis kit (TransGen Biotech, Beijing, China) according to the manufacturer’s instructions. Quantitative real-time PCR was performed with TransStart^®^ Green qPCR SuperMix (TransGen Biotech, China). The conditions for the real-time PCR reaction were as follows: 94 °C, 30 s; 94 °C, 5 s; 60 °C, 30 s; 40 cycles. The primers for this research are listed in Supplementary Material Table S1.

### 3.5. Enzyme-Linked Immunosorbent Assay (ELISA)

TNF-α and IL-1β in the cell culture supernatant were measured using commercially available ELISA kits (Biolegend, Waltham, MA, USA). Briefly, RWA264.7 cells were pre-treated with compounds for 6 h and then cells were washed with cold PBS three times. Next, fresh DMEM medium with LPS (1 μg/mL) was added into the plate. After 24 h, cell culture supernate was collected and centrifuged for 10 min (12,000× *g*, 4 °C). TNF-α and IL-1β were detected according to the manufacturer’s instructions. Specifically, the plate was washed 4 times with 300 μL of 1× Wash Buffer before adding the samples. Next, 50 μL of cell culture supernate was added into the appropriate wells and then the plate was incubated at room temperature for 2 h. After that, the plate was washed 4 times with 1× Wash Buffer, and 100 μL of Mouse TNF-α Detection Antibody solution was added into the well and incubate at room temperature for 1 h. Wells containing mouse TNF-α should turn blue in color. Finally, absorbance was read at 450 nm within 30 min.

### 3.6. Animals

C57BL/6J mice (male) were purchased from Vital River Laboratories (Beijing, China). All procedures were conducted according to the Nankai University Guidelines on Animal Care, and the experimental protocol was approved by the Institutional Animal Protection Committee of the Nankai University (Tianjing, China, NO. 2022-SYDWLL-000342). Animals were sheltered under standard conditions with food and water provided ad libitum. Experiments were conducted on 8-week-old male mice, randomly divided into 4 groups: Control (Con), ALI (bleomycin), Dex (dexamethasone) and compound **6**. For bleomycin-induced lung injury, mice were sedated, and a single dose of intratracheal bleomycin (4 mg/kg; Selleckchem, Houston, TX, USA) in sterile PBS. 6 was administered daily for 14 days via intraperitoneal injections (i.p) (100 μL/mouse of 10 mg/mL **6** in PBS). Dexamethasone (2 mg/kg) was administered orally. The mice were monitored and their weight was measured daily. After 14 days, the mice were killed with anesthetic overdose and exsanguinated by cutting the abdominal aorta before the collection of lungs.

### 3.7. Mice Survival Rates and Body Weight

Body weight variations were calculated from the measured weights at each time point (days 1–14), including before the sacrifice of the mice reaching the endpoints. To avoid a potential bias by studying the animals with the better outcomes, animals reaching endpoints were included in the calculation of the weight variation and survival rates.

### 3.8. Studies on the Release of Prodrugs into Active Drugs

A total of 10 mg of compound **6** was dissolved in 10 mL MeCN. A 0.5 mL volume of compound **6** solution was placed in 49.5 mL of HEPES at pH 7.4. The tubes were then incubated in a bath incubator at 37 °C. Samples were removed after 10 s, and the concentration of compound **6** and **3** was analyzed by HPLC.

### 3.9. Aqueous Solubility Measurement

PTL, MCL and compounds **1**–**6** (0.1, 1, 10 mg) were dissolved in deionized water (pH = 7.0) until the solution was clear. The assays were measured at least in duplicate.

## 4. Statistical Analysis

Each experiment was performed three times. One-Way ANOVA was performed to analyze the significance level by GraphPad Prism 9.0 software. *p* < 0.05 was considered to be statistically significant.

## 5. Conclusions

In conclusion, a series of ferulic acid-parthenolide (FA-PTL) or ferulic acid-micheliolide (FA-MCL) hybrids were synthesized and evaluated for their anti-inflammation activities in RAW264.7 cell lines. Compound **6** showed the potential activity against proinflammatory cytokines in the RAW264.7 cell line with IC_50_ value of 6.95 μM, which demonstrated a 2-fold decline in cytotoxic effect compared to that of the parent compound **3**. Moreover, it was more soluble than the reference compound **3**. The preliminary research into **6** indicated that compound **6** could significantly reduce inflammation of RAW264.7 cells induced by LPS. Further investigation revealed that compound **6** could significantly decrease the acute lung injury induced by bleomycin. On the basis of these results, compound **6** might be considered as a promising candidate for further evaluation as a potential anti-ALI drug.

## Figures and Tables

**Table 1:** IC_50_ of several anti-inflammatory compounds.

Entry	Compounds	MW	IC_50_ (μM)
1	FA	194.19	>100
2	MCL	248.14	9.03 ± 0.16
3	PTL	248.32	5.35 ± 0.06
4	**1**	424.18	6.14 ± 0.09
5	**2**	469.25	19.58 ± 0.21
6	**3**	440.18	3.04 ± 0.08
7	**4**	484.26	38.39 ± 1.14
8	**5**	661.27	28.80 ± 0.21
9	**6**	677.25	6.95 ± 1.09

All values are the means of three independent experiments. MW, molecular weight; IC_50_, half maximal inhibitory concentration.

**Table 2:** The aqueous solubility of compounds **1**–**6** ^a^.

Compounds	Aqueous Solubility IC_50_ (μg/mL)
	pH = 7.0
PTL	50
MCL	50
**1**	<10
**2**	<10
**3**	<10
**4**	<10
**5**	2500
**6**	2500

^a^: The assays were measured at least in duplicate.

## Data Availability

Data are contained within the article and supplementary materials.

## Supplementary Materials

The following supporting information can be downloaded at: https://www.mdpi.com/article/10.3390/molecules29050936/s1. Figures S1–S26: the ^1^H and ^13^C NMR spectra of compounds (**7**, **9**, **1**, **10**, **2**, **5**, **3**, **12**, **6**, **13**, **14**, **15**, **16**, and **4**); Table S1: Primers for qRT-PCR.
