# Supplementary material for: Synthesis and Anti-Inflammatory Activity of Ferulic Acid-Sesquiterpene Lactone Hybrids
Source: Molecules. 2024 Feb 21;29(5):936. doi: 10.3390/molecules29050936 (PMC10933920; doi:10.3390/molecules29050936)
Supplement: Supplementary file 1 [file molecules-29-00936-s001.zip › molecules-2837372-supplementary.pdf]

Supporting Information

Synthesis and Anti-Inflammatory Activity of Ferulic Acid-Sesquiterpene Lactone Hybrids

Xiyan Duan [1,*], Ning Liu [2], Ke Lv [3,*], Junqi Wang [1], Mingyue Li [4], Yanwei Zhang [5], Xiaoguang Huo [5], Shiqi Bao [5], Zhuo Shen [5] and Xuemei Zhang [5]

1 School of Chemistry & Chemical Engineering, Henan University of Science and Technology, Luoyang 471003, China; 17513624203@163.com
2 School of Nursing, Henan University of Science and Technology, Luoyang 471003, China; 9943548@haust.edu.cn
3 The State Key Laboratory of Medicinal Chemical Biology & College of Chemistry, Nankai University, Tianjin 300071, China
4 College of Pharmacy, Nankai University, Tianjin 300071, China; lmy1137534123@163.com
5 Accendatech Company, Ltd., Tianjin 300384, China; zyw12130123@163.com (Y.Z.); huoxg0323@163.com (X.H.); shiqi.bao@accendatech.com (S.B.); shenzhuo@accendatech.com (Z.S.); xuemei.zhang@accendatech.com (X.Z.)
* Correspondence: duanxiyan@tju.edu.cn (X.D.); lk2387723328@gmail.com (K.L.)

Table of Contents

Figure S1 1H NMR spectra of compound 7 3
Figure S2 1H NMR spectra of compound 9 4
Figure S3 13C NMR spectra of compound 9 4
Figure S4 1H NMR spectra of compound 1 5
Figure S5 13C NMR spectra of compound 1 5
Figure S6 1H NMR spectra of compound 10 6
Figure S7 13C NMR spectra of compound 10 6
Figure S8 1H NMR spectra of compound 2 7
Figure S9 13C NMR spectra of compound 2 7
Figure S10 1H NMR spectra of compound 5 8
Figure S11 13C NMR spectra of compound 5 8
Figure S12 1H NMR spectra of compound 3 9
Figure S13 1H NMR spectra of compound 12 9
Figure S14 13C NMR spectra of compound 12 10
Figure S15 1H NMR spectra of compound 6 10
Figure S16 13C NMR spectra of compound 6 11
Figure S17 1H NMR spectra of compound 13 11
Figure S18 13C NMR spectra of compound 13 12
Figure S19 1H NMR spectra of compound 14 12
Figure S20 13C NMR spectra of compound 14 13
Figure S21 1H NMR spectra of compound 15 13
Figure S22 13C NMR spectra of compound 15 14
Figure S23 1H NMR spectra of compound 16 14
Figure S24 13C NMR spectra of compound 16 15
Figure S25 1H NMR spectra of compound 4 15
Figure S26 13C NMR spectra of compound 4 15
Table S1 Primers for qRT-PCR 16

1. Spectra for compounds

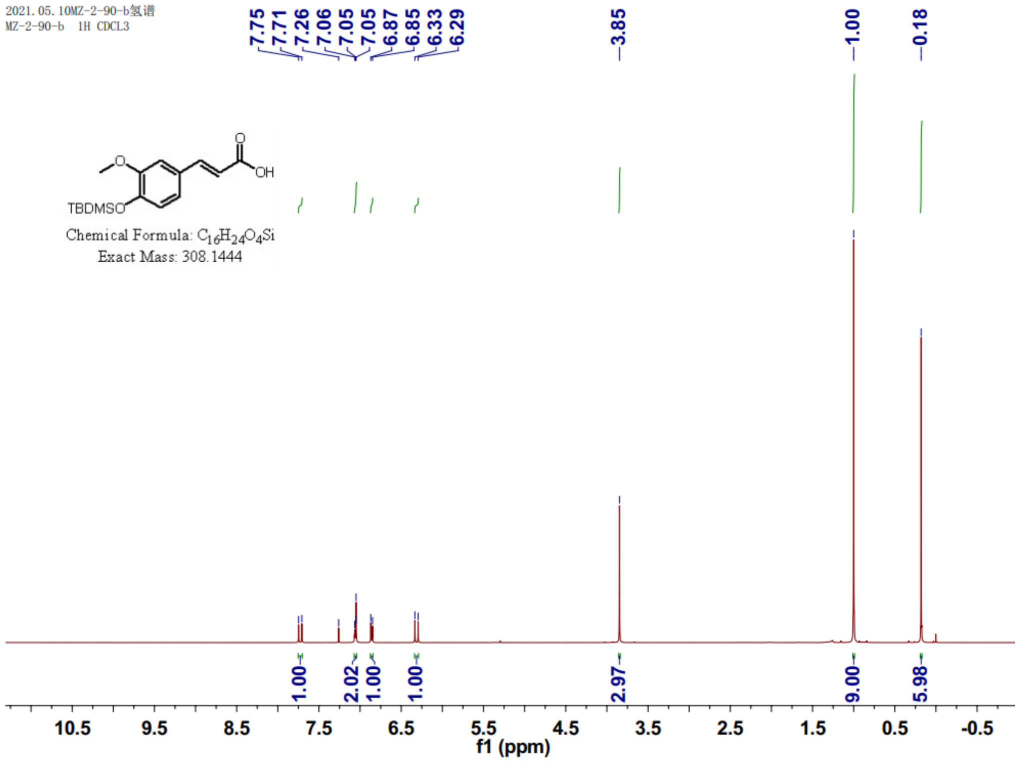

Figure S1 1H NMR spectra of compound 7

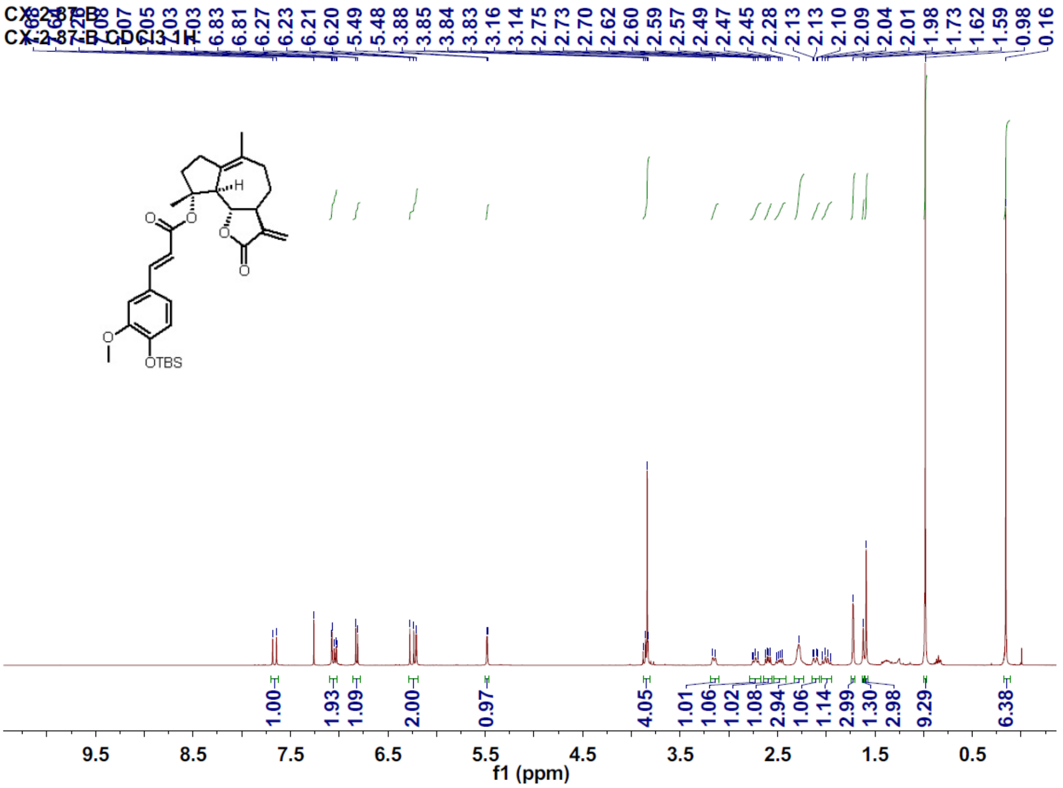

Figure S2 1H NMR spectra of compound 9

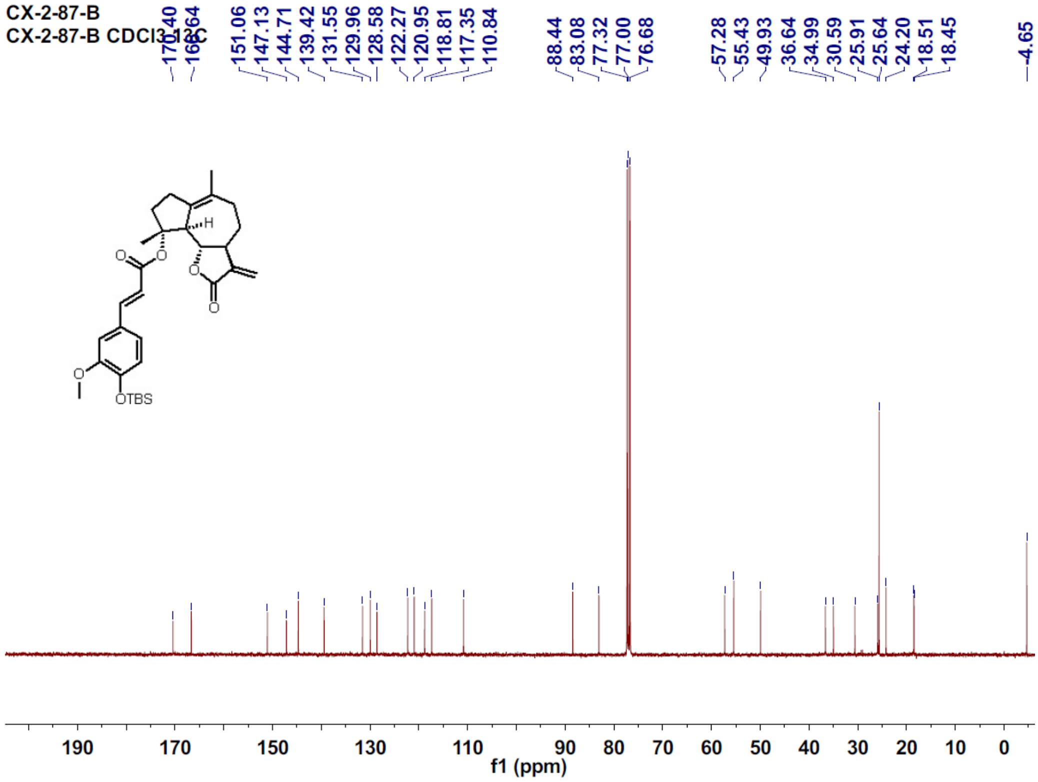

Figure S3 13C NMR spectra of compound 9

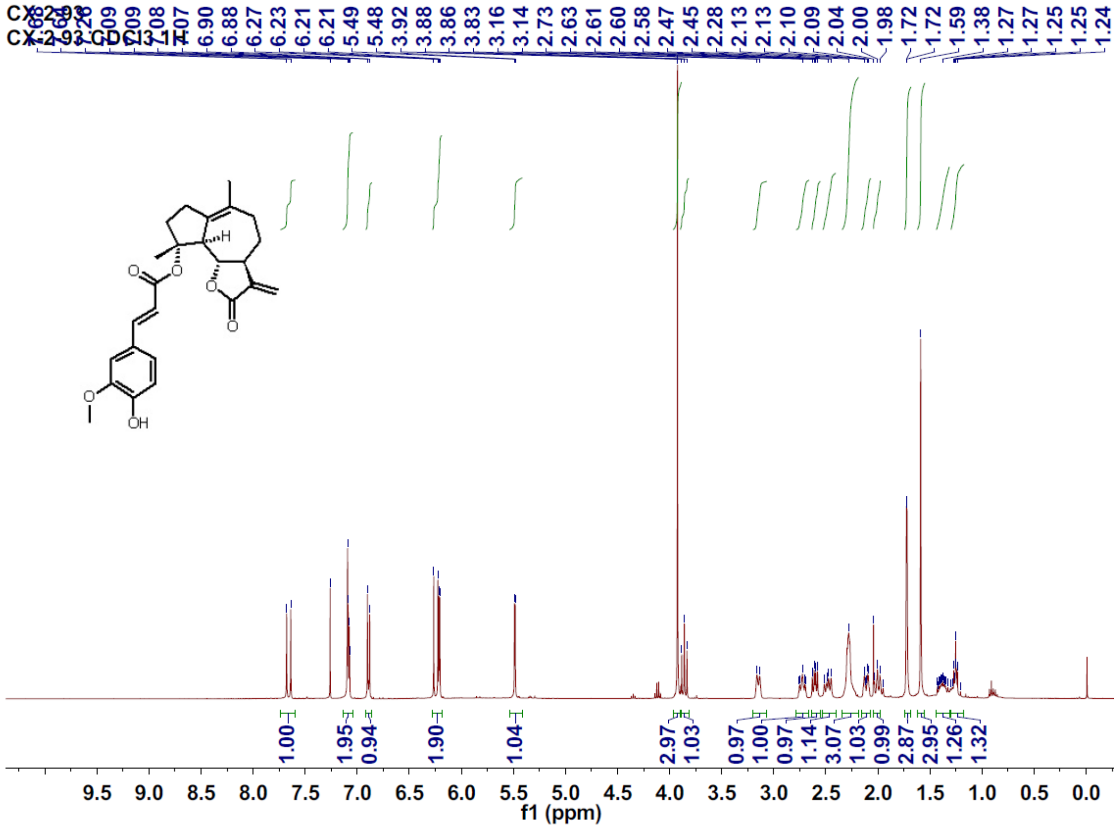

Figure S4 1H NMR spectra of compound 1

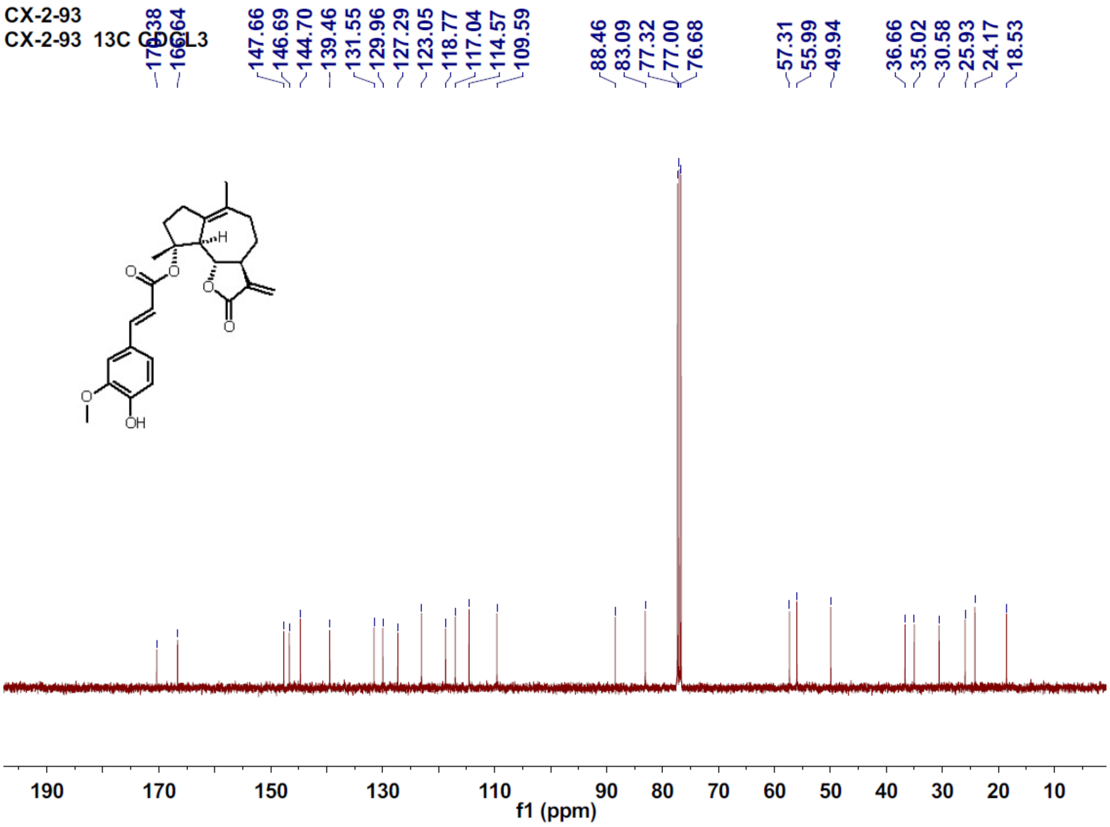

Figure S5 13C NMR spectra of compound 1

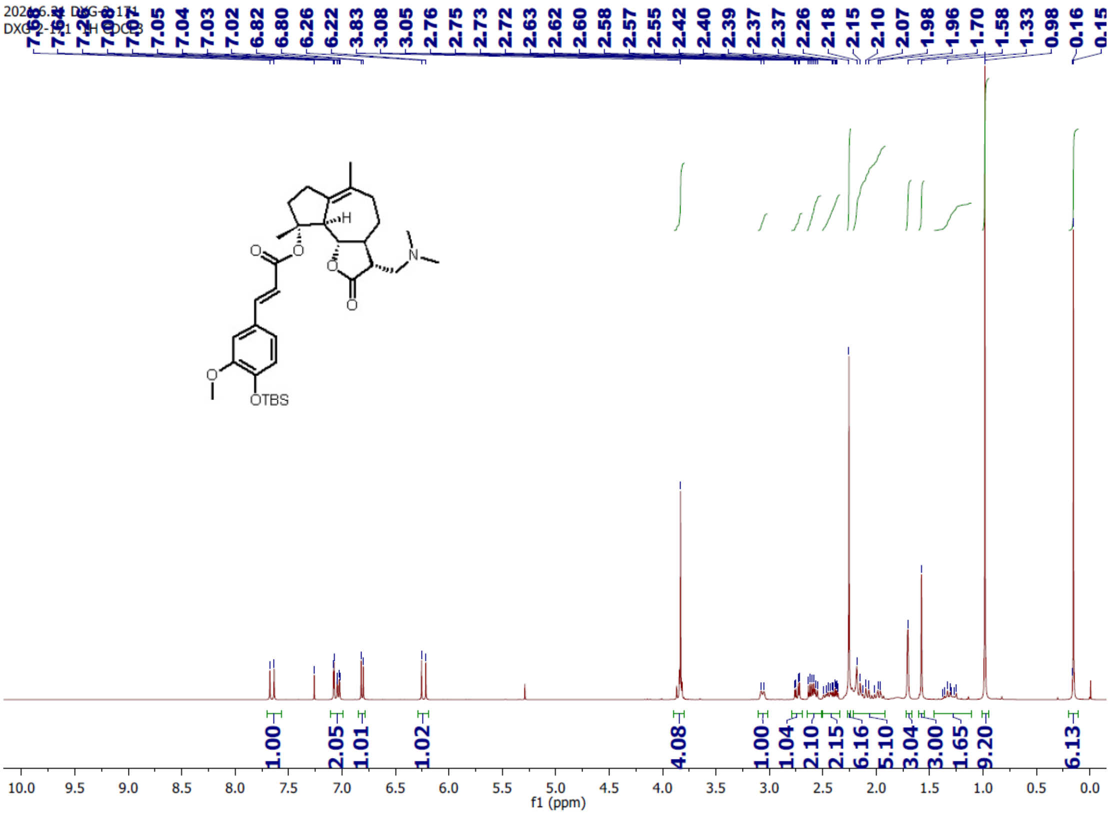

Figure S6 1H NMR spectra of compound 10

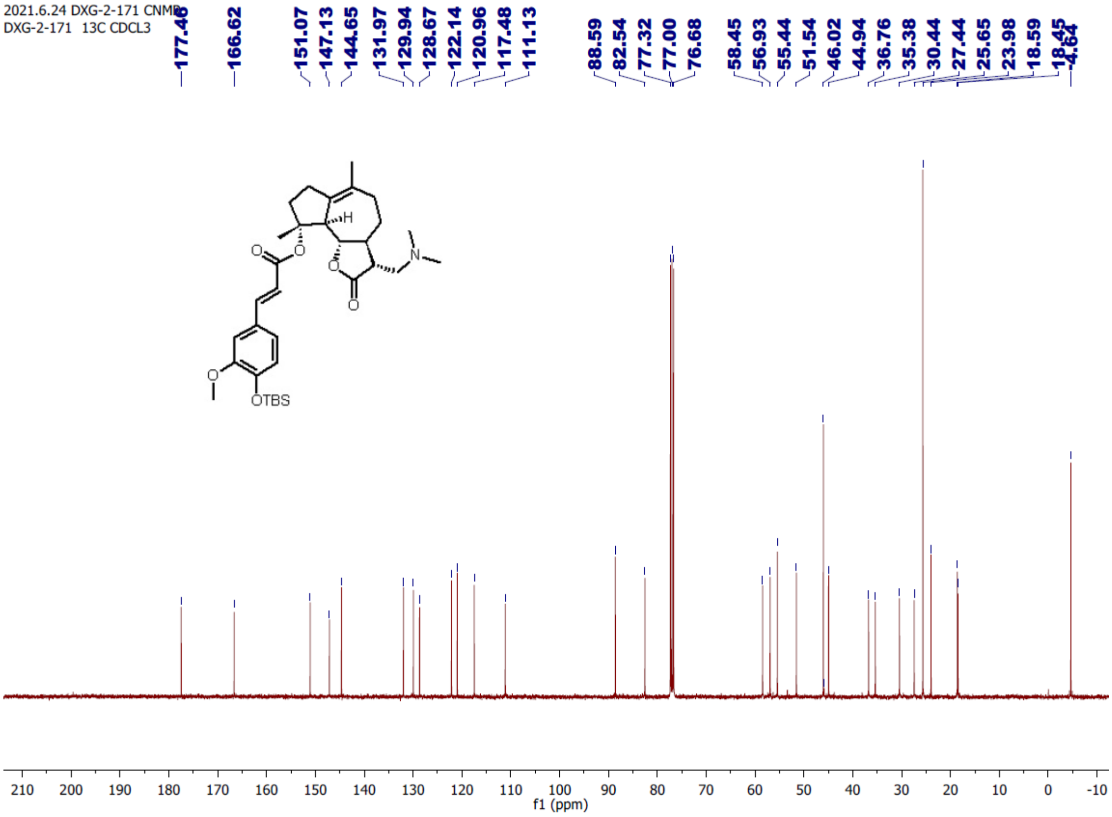

Figure S7 13C NMR spectra of compound 10

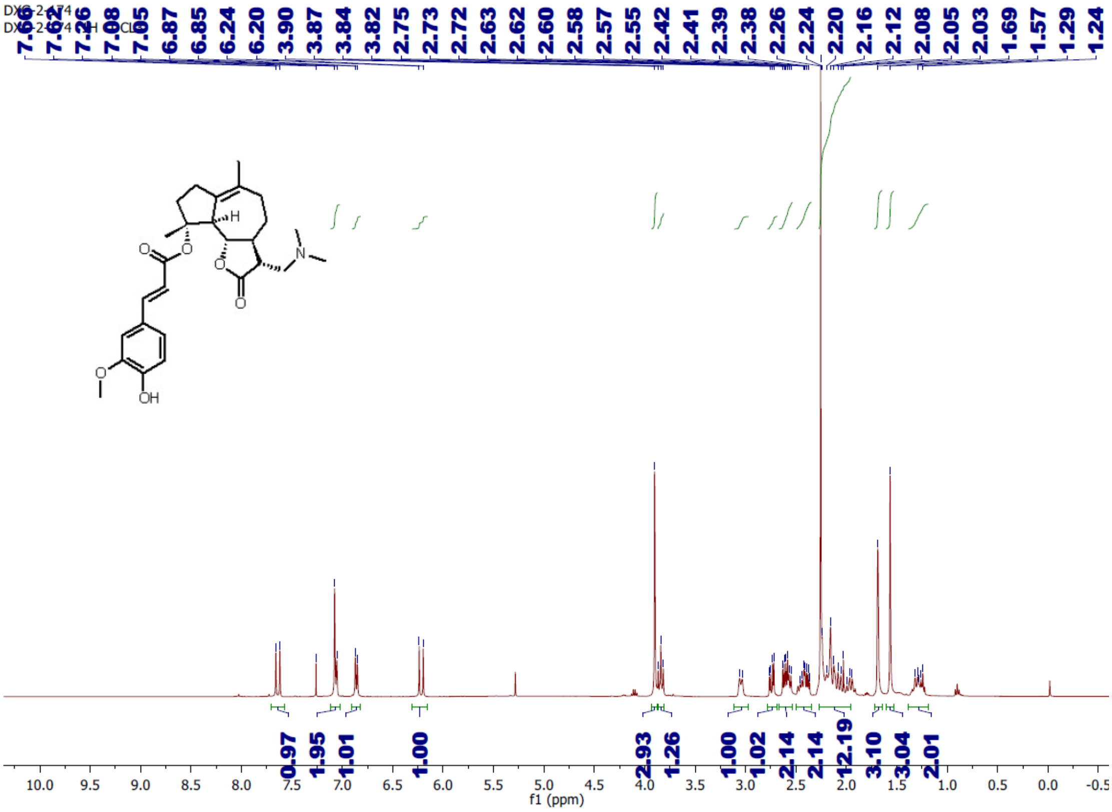

Figure S8 1H NMR spectra of compound 2

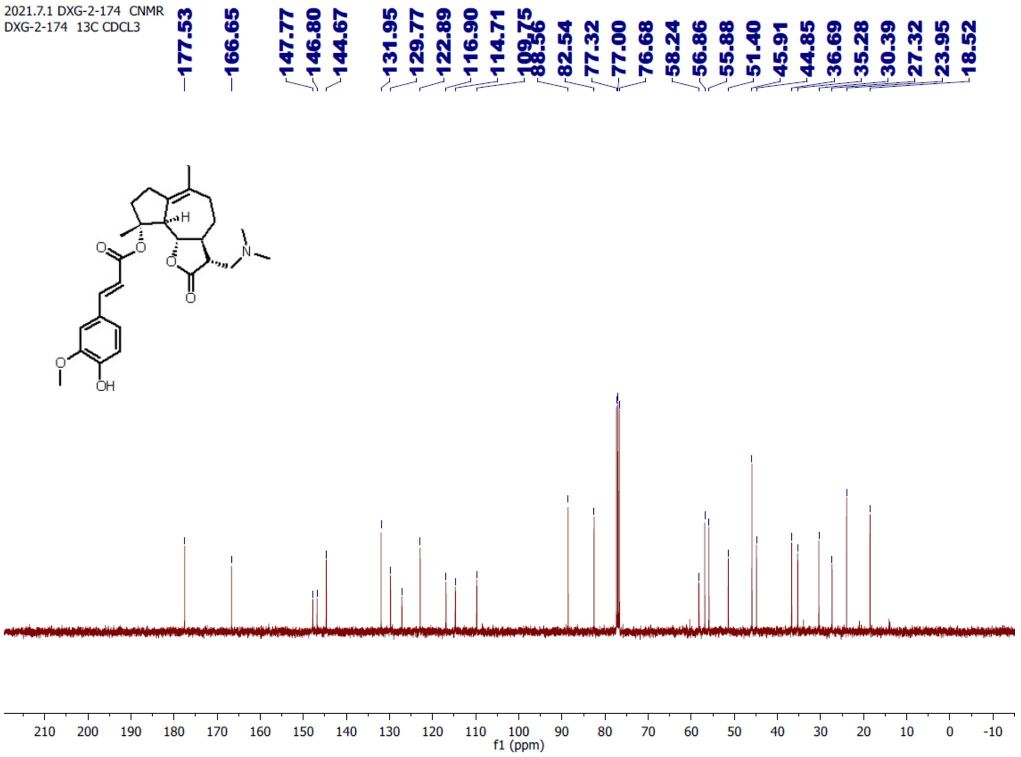

Figure S9 13C NMR spectra of compound 2

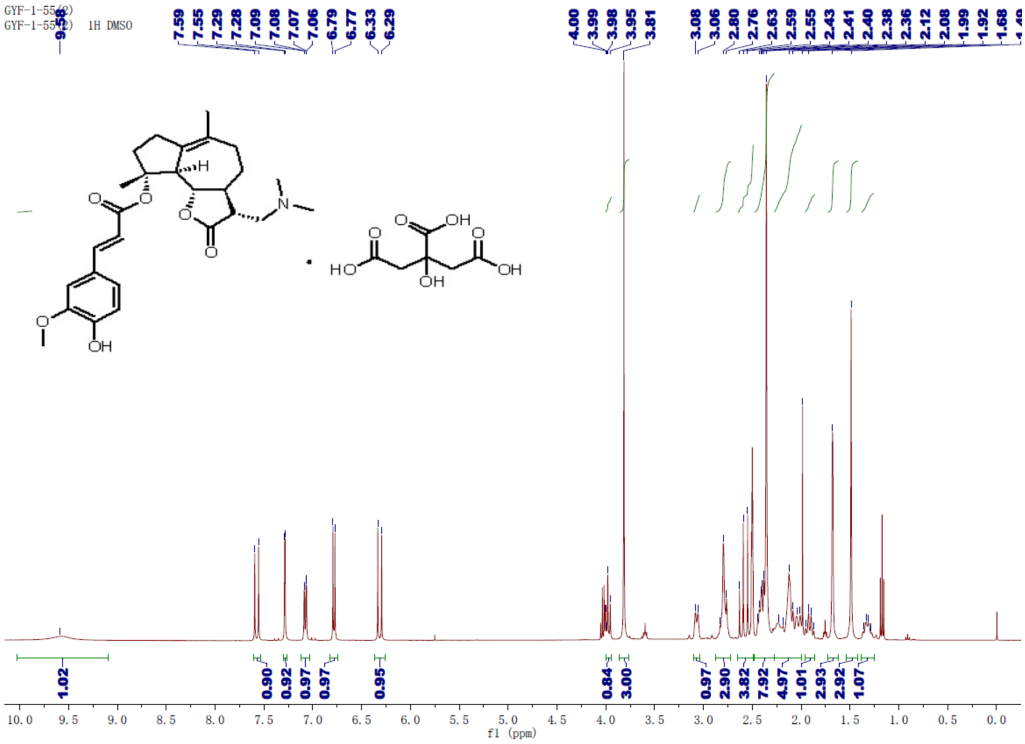

Figure S10 1H NMR spectra of compound 5

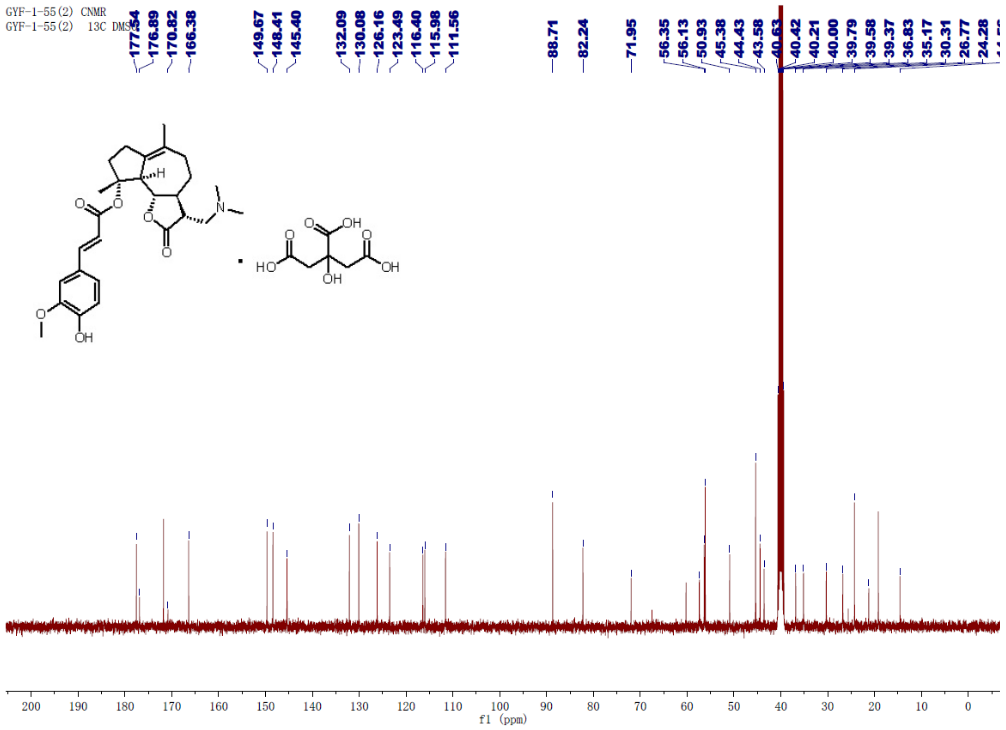

Figure S11 13C NMR spectra of compound 5

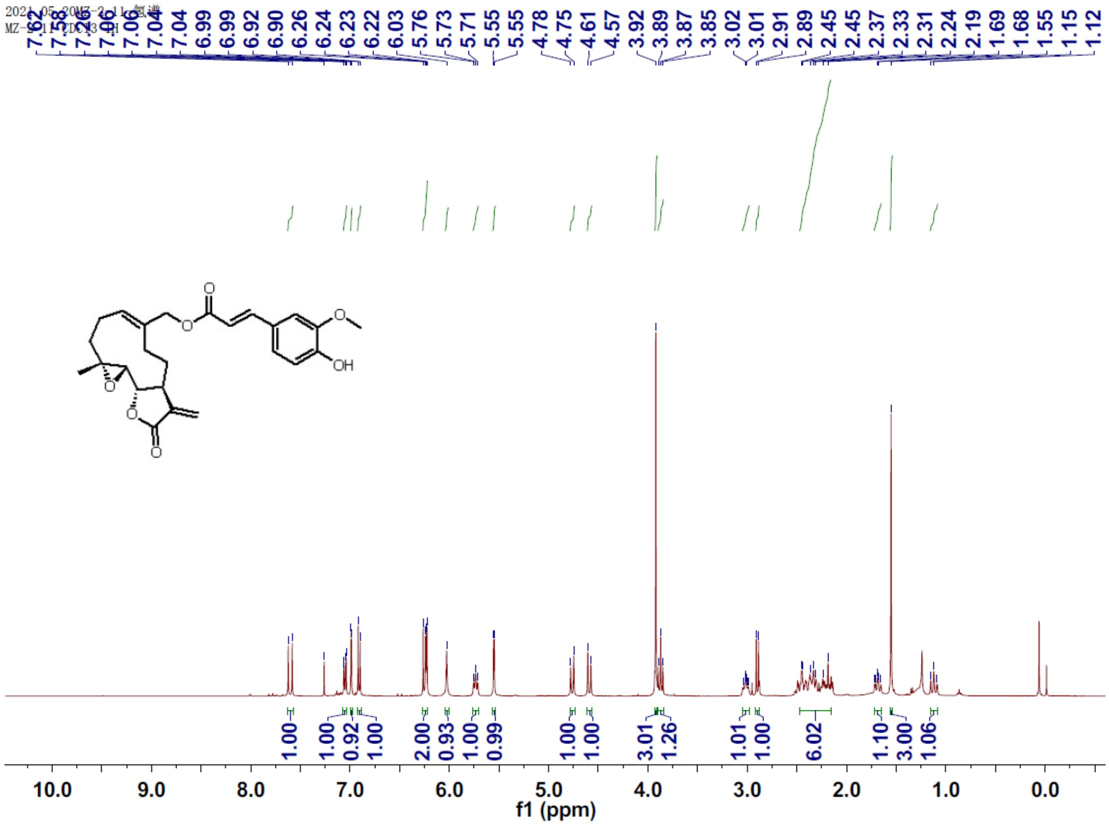

Figure S12 1H NMR spectra of compound 3

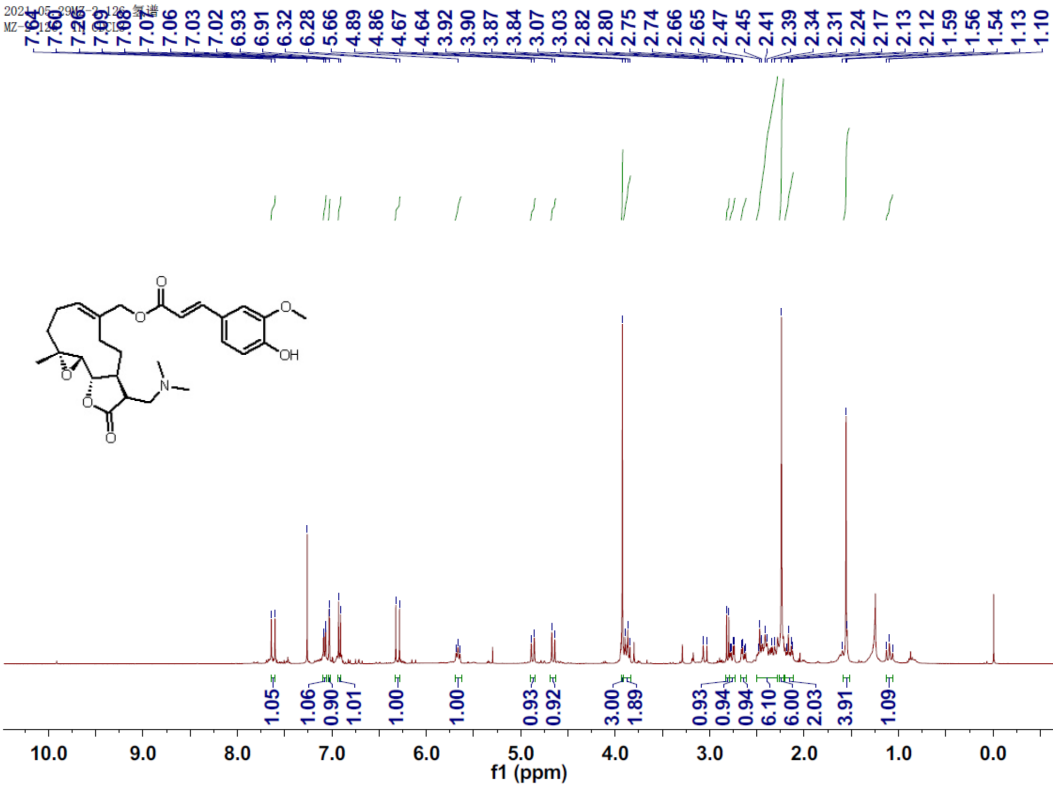

Figure S13 1H NMR spectra of compound 12

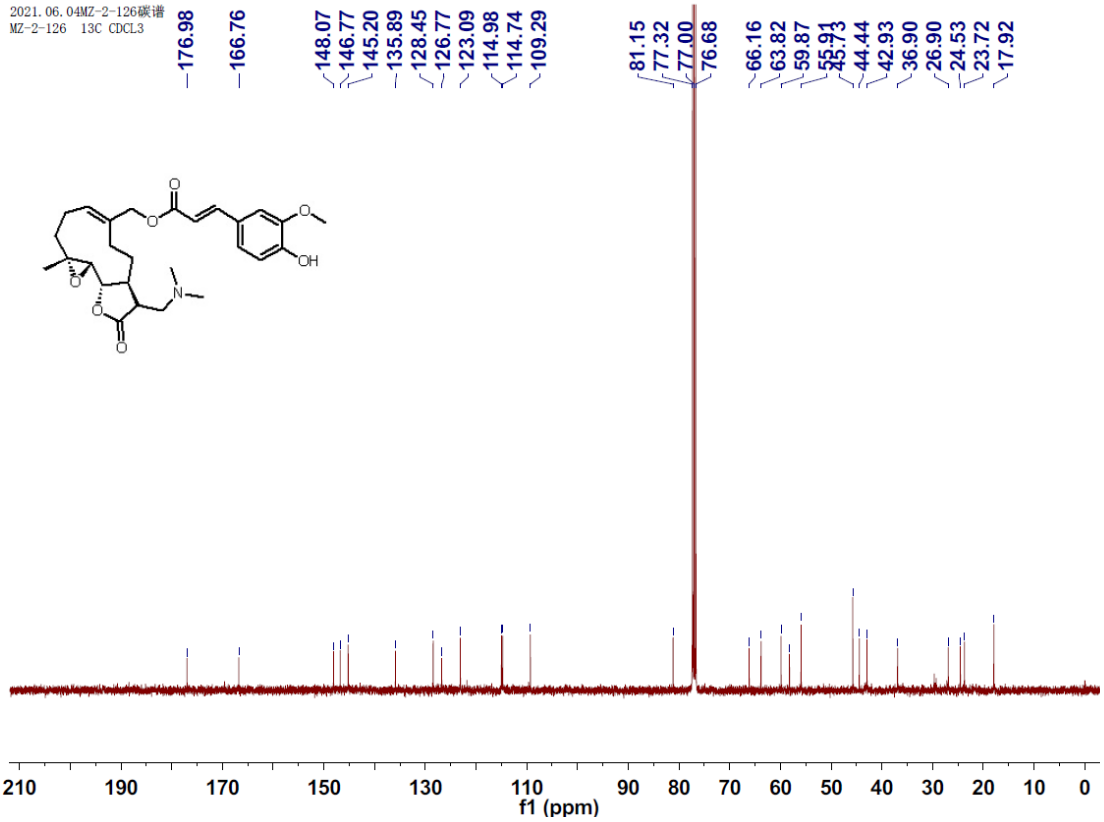

Figure S14 13C NMR spectra of compound 12

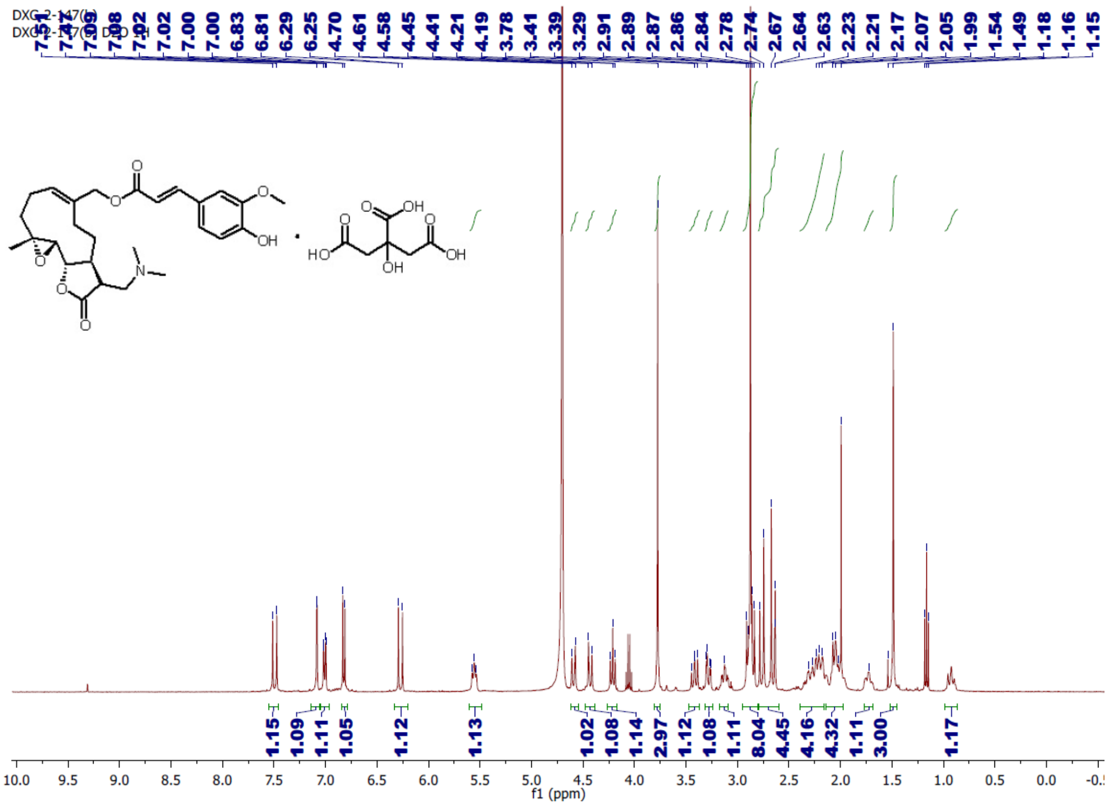

Figure S15 1H NMR spectra of compound 6

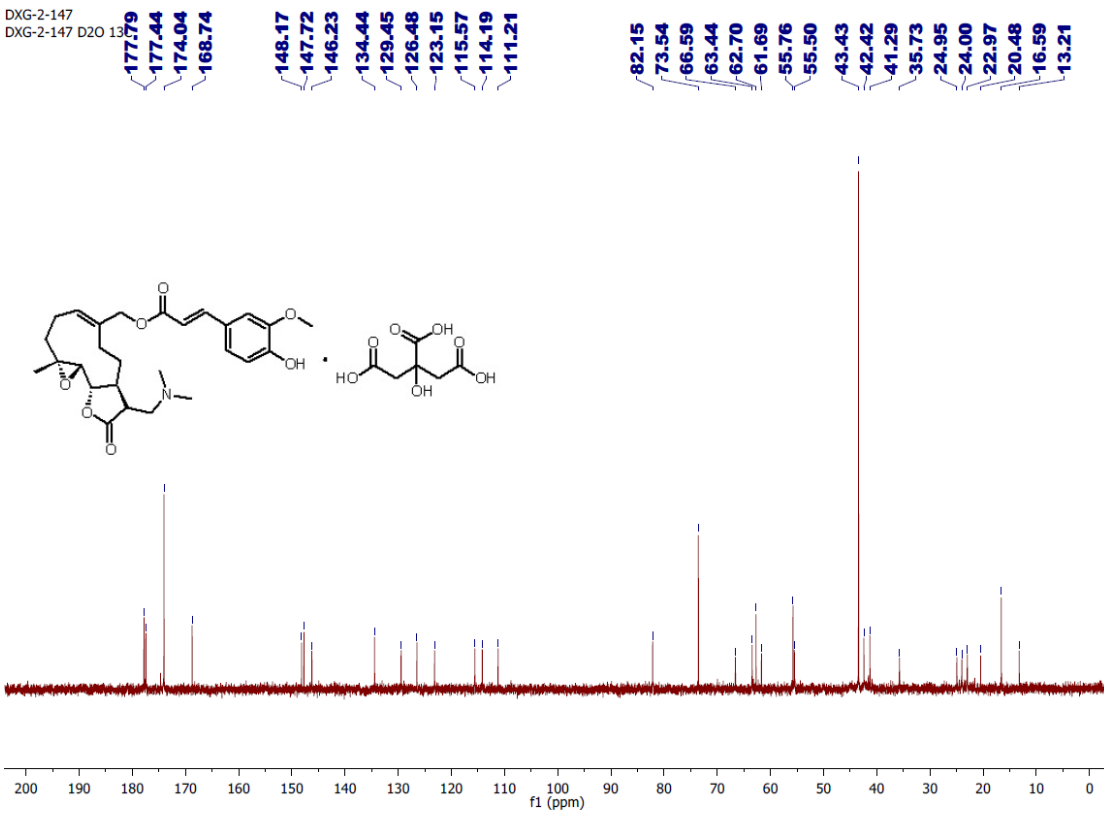

Figure S16 13C NMR spectra of compound 6

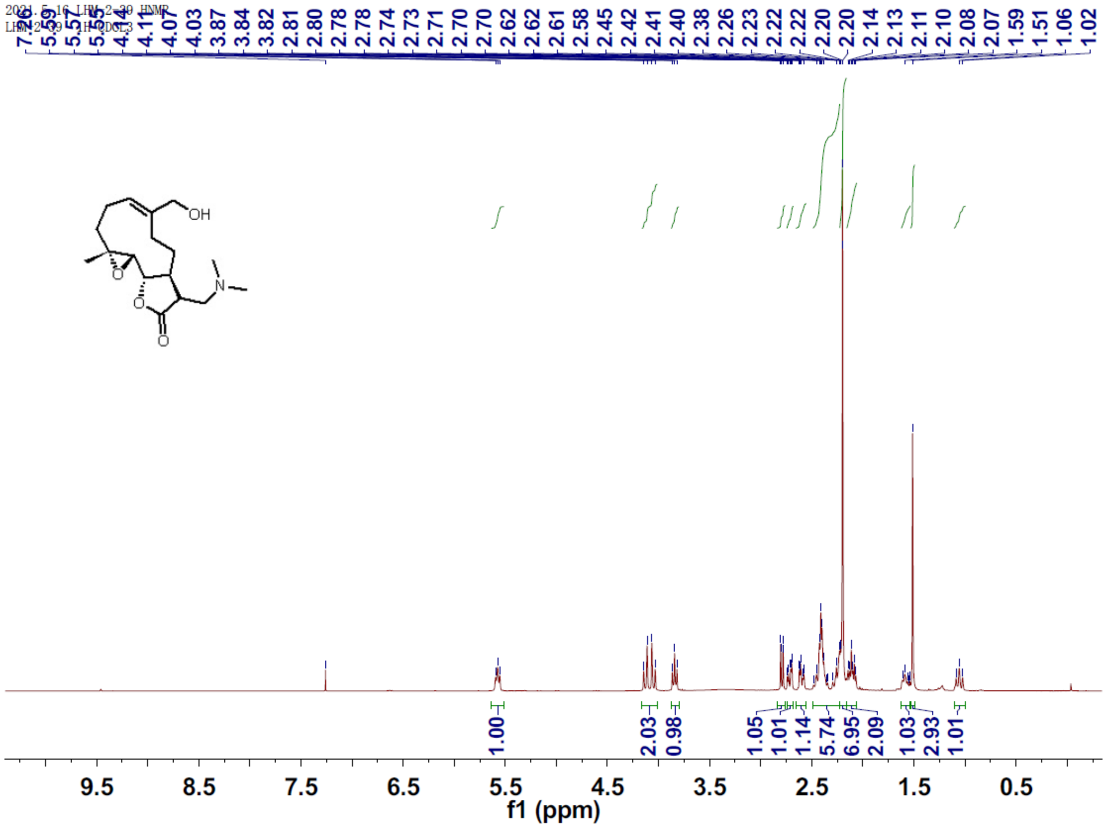

Figure S17 1H NMR spectra of compound 13

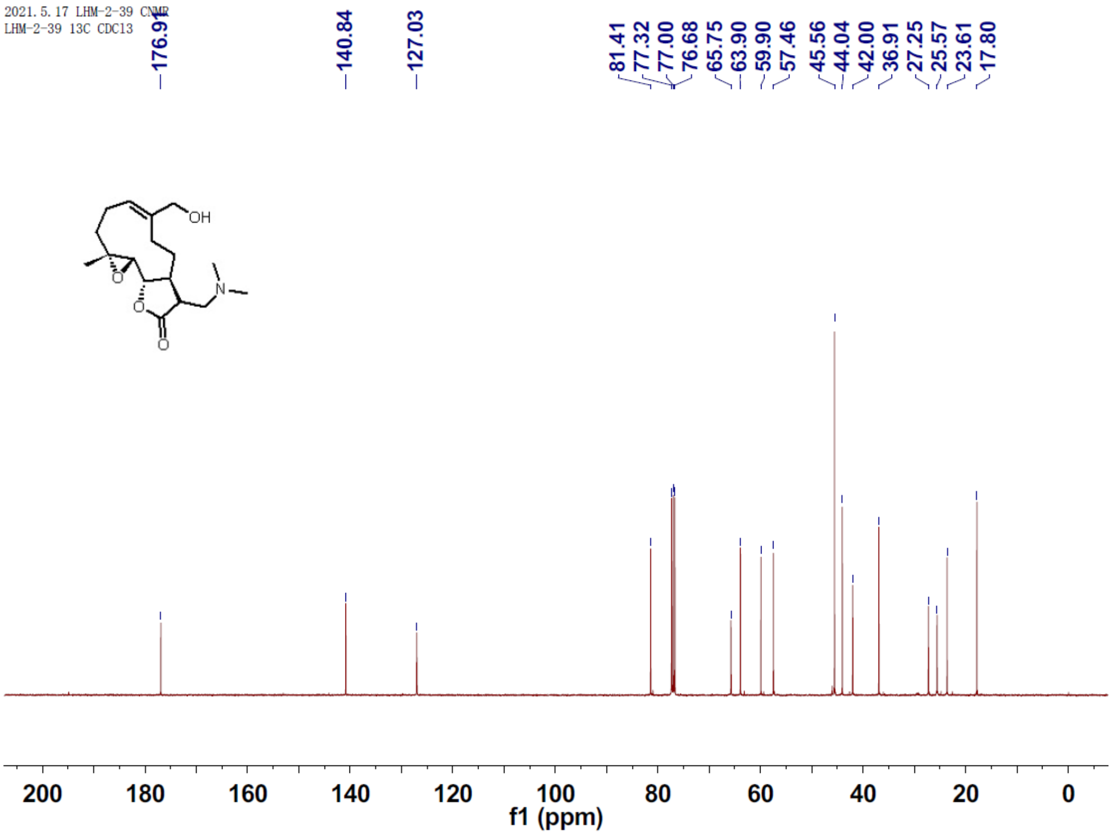

Figure S18 13C NMR spectra of compound 13

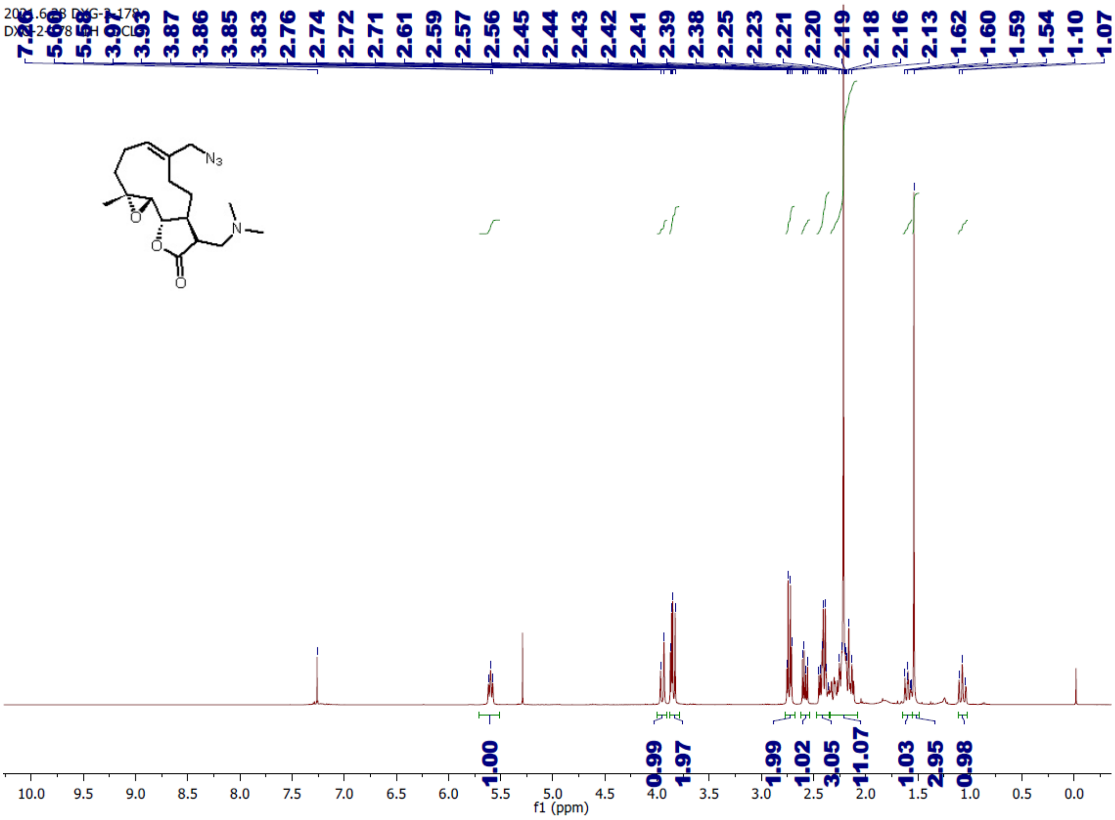

Figure S19 1H NMR spectra of compound 14

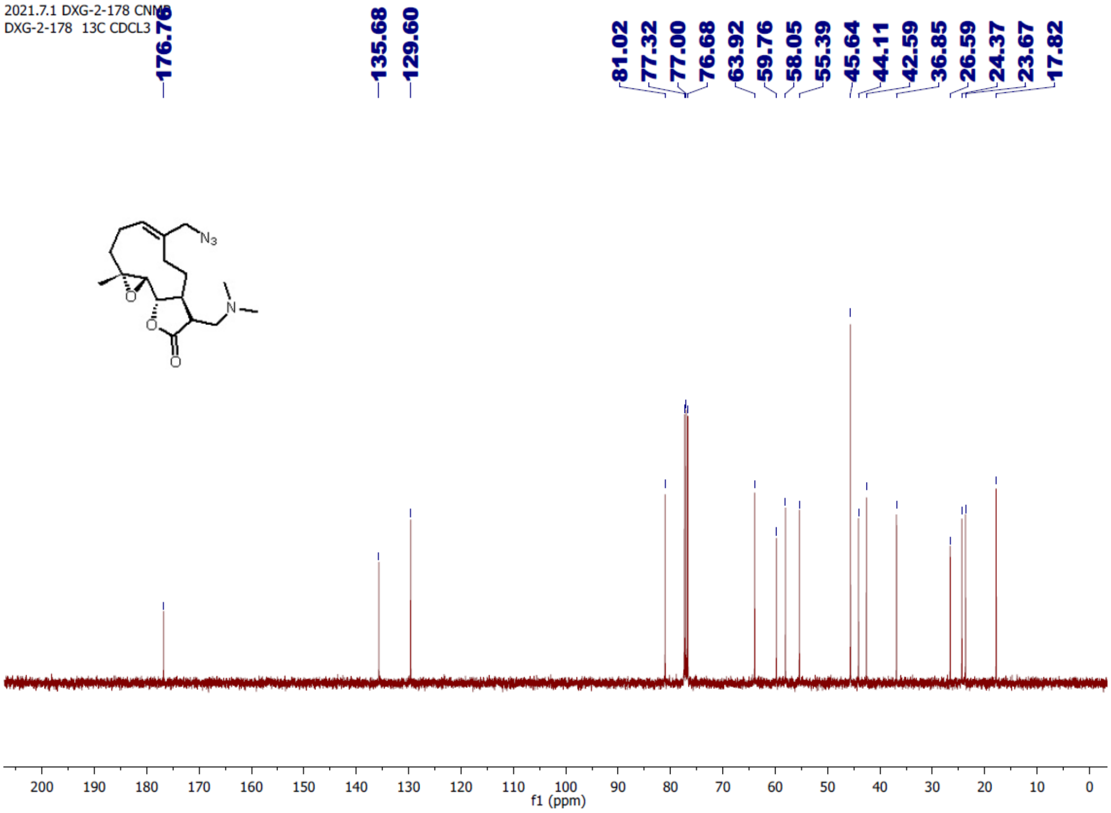

Figure S20 13C NMR spectra of compound 14

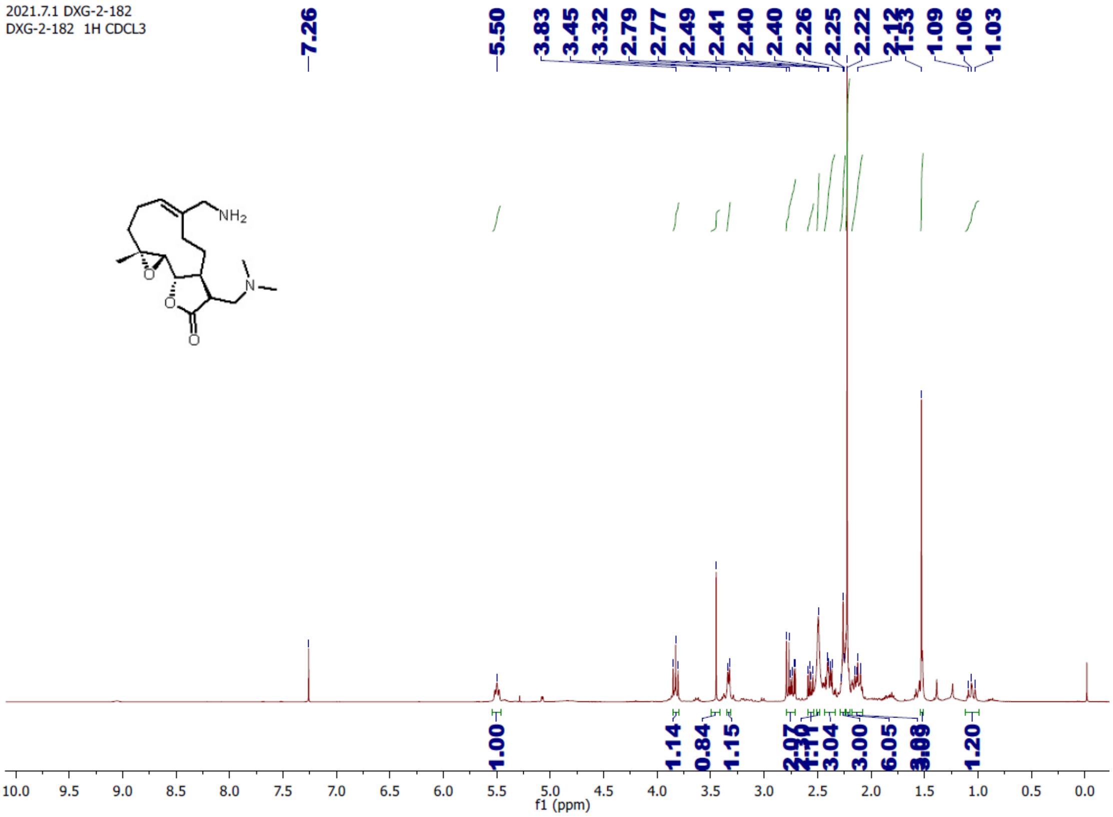

Figure S21 1H NMR spectra of compound 15

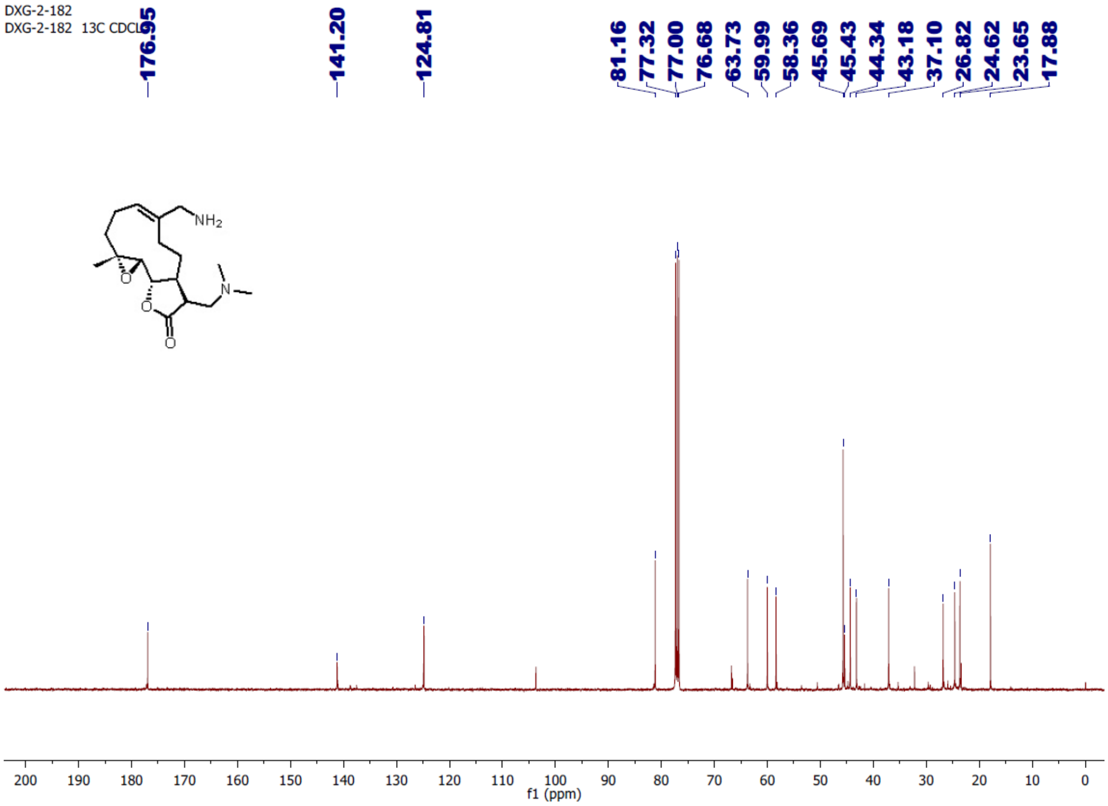

Figure S22 13C NMR spectra of compound 15

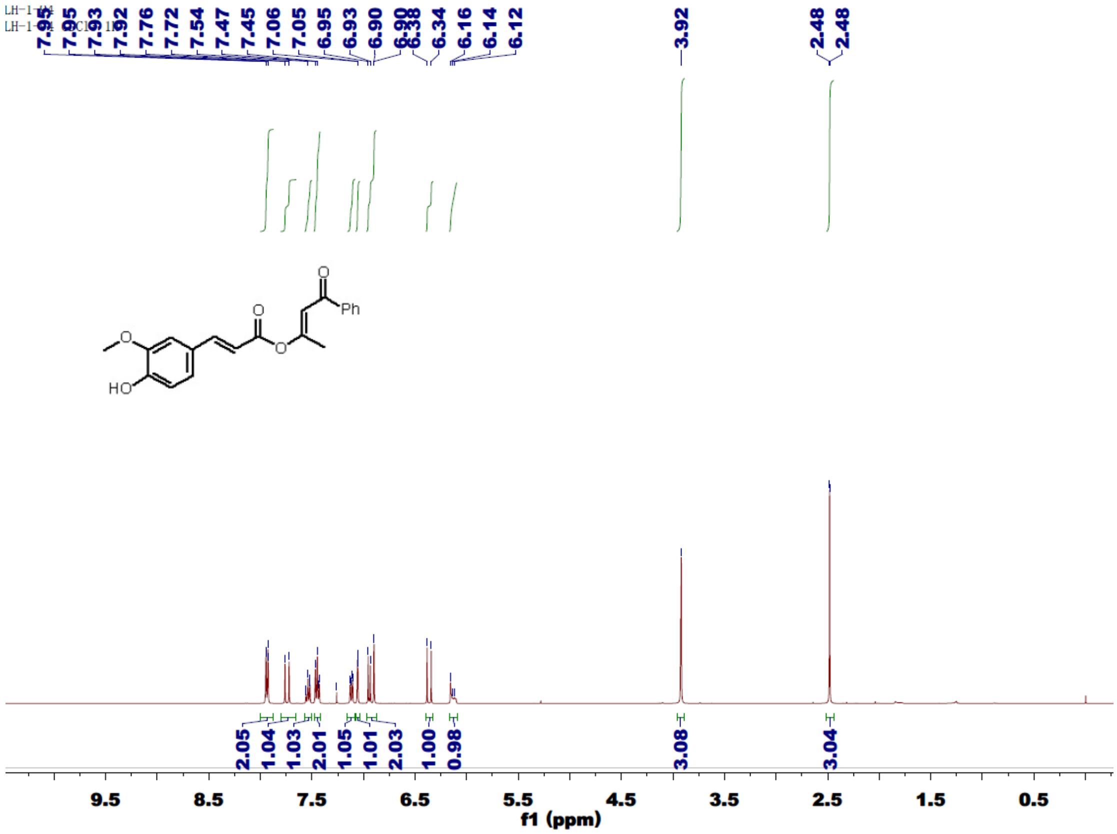

Figure S23 1H NMR spectra of compound 16

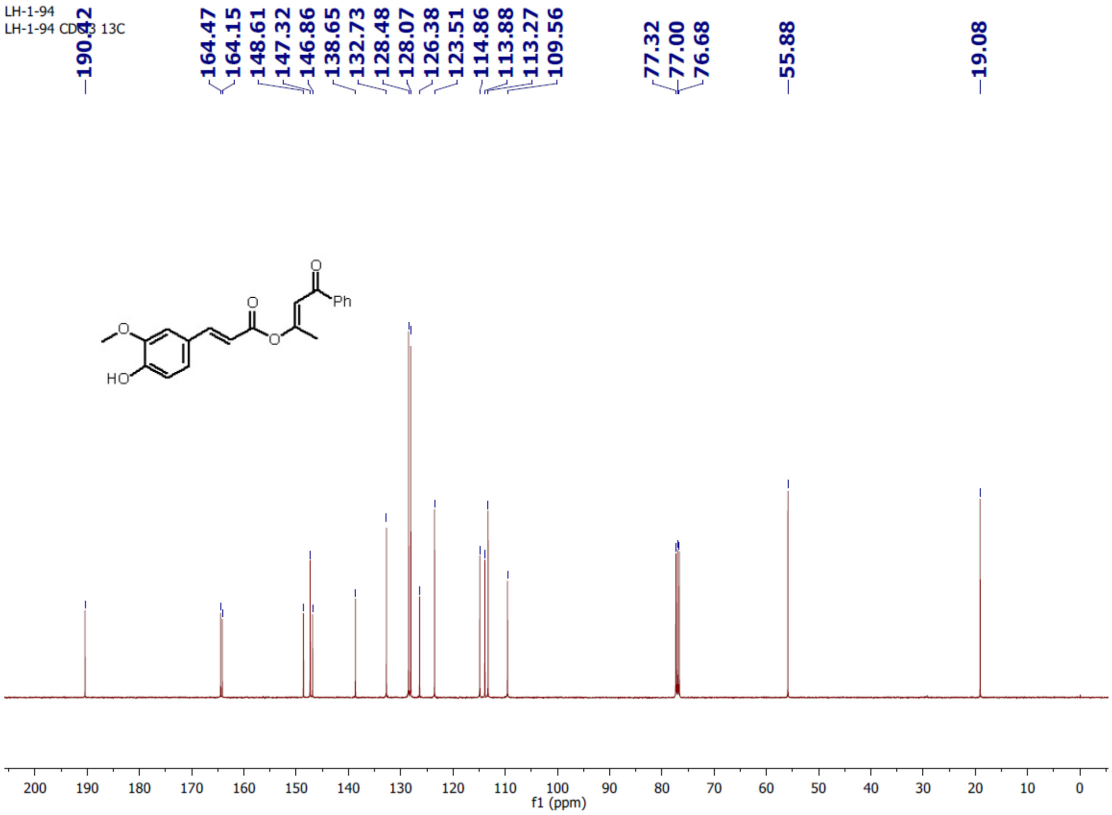

Figure S24 13C NMR spectra of compound 16

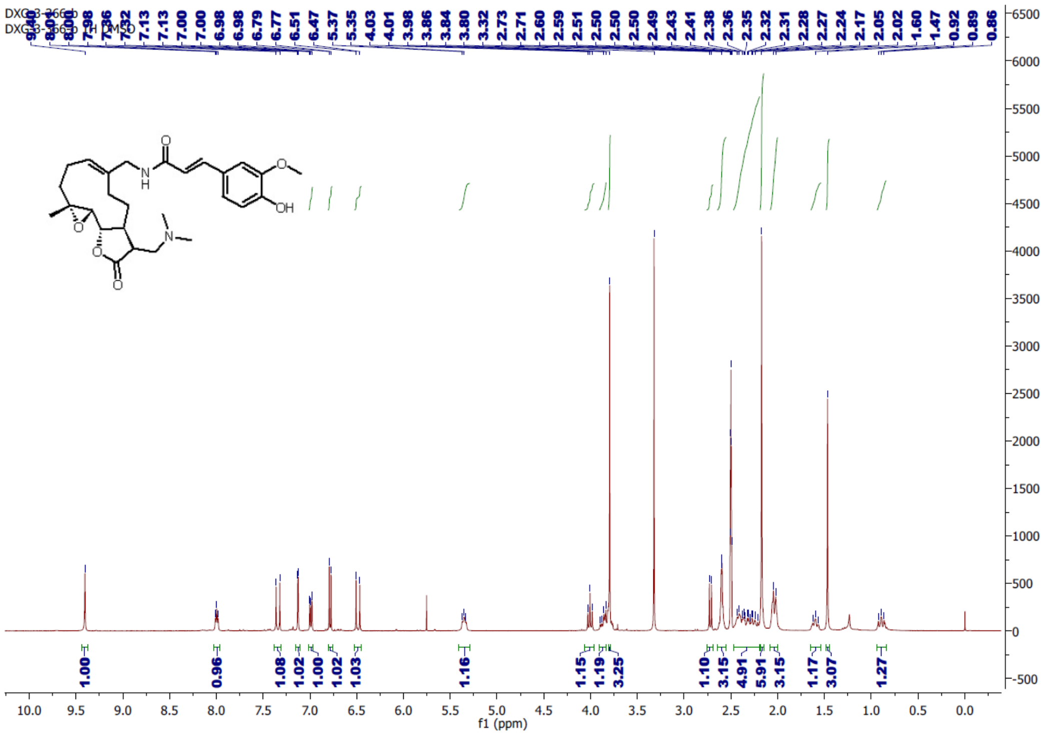

Figure S25 1H NMR spectra of compound 4

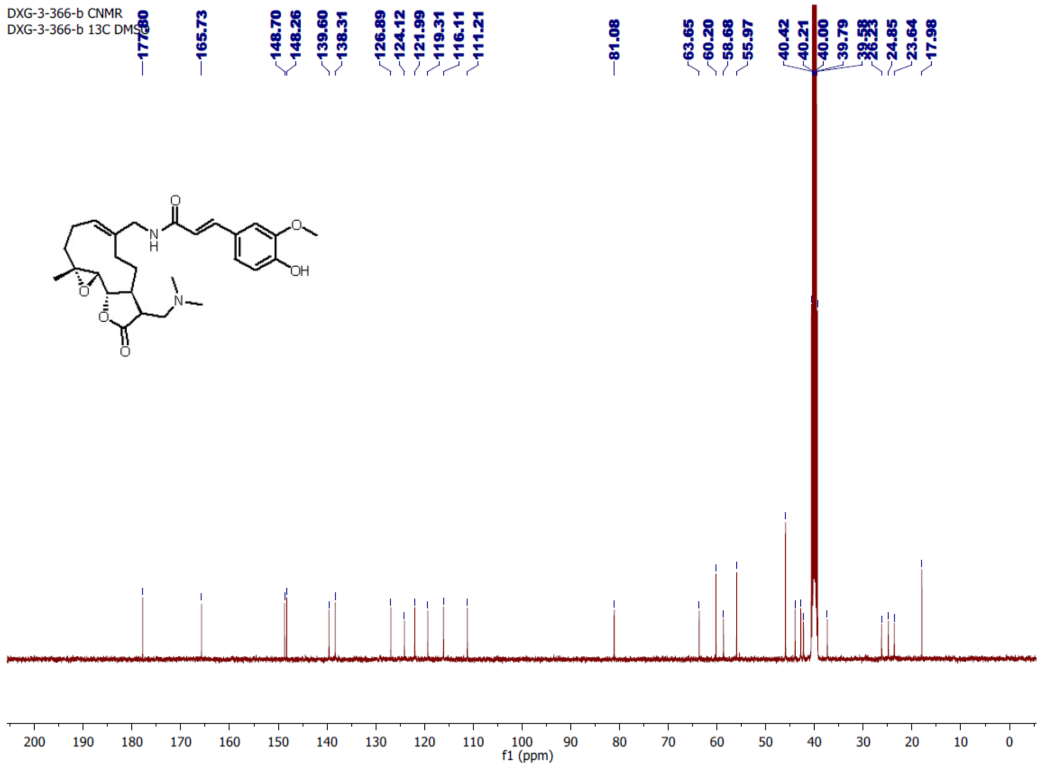

Figure S26 13C NMR spectra of compound 4

Table S1 Primers for qRT-PCR

Forward: TNF-α	GAAGATCACCTCCGCTCCCAATG
Reverse: TNF-α	ATGTTCCCGTCCACCACCTCTC
Forward: mF-IL-6	CTTCTTGGGACTGATGCTGGTGAC
Reverse: mR-IL-6	AGGTCTGTTGGGAGTGGTATCCTC
Forward: mF-IL-1β	TCGCAGCAGCACATCAACAAGAG
Reverse: mR-IL-1β	AGGTCCACGGGAAAGACACAGG
Forward: mF18S	CGCCGCTAGAGGTGAAATTCT
Reverse: mR18S	CGAACCTCCGACTTTCGTTCT
Forward: iNOS	AGGAGGAGAGAGATCCGATTTAG
Reverse: iNOS	TCAGACTTCCCTGTCTCAGTAG
